# A genome scale metabolic network for rice and accompanying analysis of tryptophan, auxin and serotonin biosynthesis regulation under biotic stress

**DOI:** 10.1186/1939-8433-6-15

**Published:** 2013-05-29

**Authors:** Palitha Dharmawardhana, Liya Ren, Vindhya Amarasinghe, Marcela Monaco, Jim Thomason, Dean Ravenscroft, Susan McCouch, Doreen Ware, Pankaj Jaiswal

**Affiliations:** Department of Botany and Plant Pathology, Oregon State University, 2082-Cordley Hall, Corvallis, OR 97331 USA; Cold Spring Harbor Laboratory, Cold Spring Harbor, NY 11724 USA; Department of Plant Breeding and Genetics, Cornell University, Ithaca, NY USA

**Keywords:** Rice, Oryza sativa, Metabolic network, Diurnal, Tryptophan biosynthesis, Serotonin biosynthesis, Auxin biosynthesis, Biotic stress, RiceCyc, Gene regulation

## Abstract

**Background:**

Functional annotations of large plant genome projects mostly provide information on gene function and gene families based on the presence of protein domains and gene homology, but not necessarily in association with gene expression or metabolic and regulatory networks. These additional annotations are necessary to understand the physiology, development and adaptation of a plant and its interaction with the environment.

**Results:**

RiceCyc is a metabolic pathway networks database for rice. It is a snapshot of the substrates, metabolites, enzymes, reactions and pathways of primary and intermediary metabolism in rice. RiceCyc version 3.3 features 316 pathways and 6,643 peptide-coding genes mapped to 2,103 enzyme-catalyzed and 87 protein-mediated transport reactions. The initial functional annotations of rice genes with InterPro, Gene Ontology, MetaCyc, and Enzyme Commission (EC) numbers were enriched with annotations provided by KEGG and Gramene databases. The pathway inferences and the network diagrams were first predicted based on MetaCyc reference networks and plant pathways from the Plant Metabolic Network, using the Pathologic module of Pathway Tools. This was enriched by manually adding metabolic pathways and gene functions specifically reported for rice. The RiceCyc database is hierarchically browsable from pathway diagrams to the associated genes, metabolites and chemical structures. Through the integrated tool OMICs Viewer, users can upload transcriptomic, proteomic and metabolomic data to visualize expression patterns in a virtual cell. RiceCyc, along with additional species-specific pathway databases hosted in the Gramene project, facilitates comparative pathway analysis.

**Conclusions:**

Here we describe the RiceCyc network development and discuss its contribution to rice genome annotations. As a case study to demonstrate the use of RiceCyc network as a discovery environment we carried out an integrated bioinformatic analysis of rice metabolic genes that are differentially regulated under diurnal photoperiod and biotic stress treatments. The analysis of publicly available rice transcriptome datasets led to the hypothesis that the complete tryptophan biosynthesis and its dependent metabolic pathways including serotonin biosynthesis are induced by taxonomically diverse pathogens while also being under diurnal regulation. The RiceCyc database is available online for free access at http://www.gramene.org/pathway/.

**Electronic supplementary material:**

The online version of this article (doi:10.1186/1939-8433-6-15) contains supplementary material, which is available to authorized users.

## Background

The growing number of sequenced plant genomes has opened up immense opportunities to study biological processes related to plant physiology, growth and development, and biotic and abiotic stress at the cellular and whole plant level using a novel systems-level approach. Arabidopsis and rice (*Oryza sativa*) continue to be the flagship plant models and share a significant fraction of reported literature on gene functions and phenotypes associated with plant development and metabolism. Therefore, newly sequenced plant genomes and transcriptomes depend heavily on rice and Arabidopsis genome annotations for projecting genome, gene product and pathway annotations. In the majority of cases gene product annotations involve InterPro (Hunter, Jones et al. 2012) and Gene Ontology (GO) assignments, which are often enriched by the addition of annotations from *Arabidopsis* and rice based on sequence homology. Depending on the biological question these annotations are further evaluated to model the metabolic (Urbanczyk-Wochniak and Sumner 2007; Dal'Molin, Quek et al. Quek et al. 2010; Zhang, Dreher et al. Dreher et al. 2010a; Saha, Suthers et al. Suthers et al. 2011; Monaco, Sen et al. Sen et al. 2012; Monaco, Sen et al. Sen et al. 2013), regulatory (Yun, Park et al. 2010) and co-expressed networks (Childs, Davidson et al. 2011; Ficklin and Feltus 2011) leading to novel discoveries of genes and enzymes regulating important agronomic traits (Kuroha, Tokunaga et al. 2009; Tokunaga, Kojima et al. Kojima et al. 2012).

In order to respond to and survive environmental challenges, plants, as sessile organisms, have developed a multitude of anatomical, morphological, growth habit and developmental adaptations that are based on underlying genetic variation. Gene duplication and alternative splicing of gene transcripts, commonly seen in plant species, provide plasticity and “sub-functionalization” that can contribute to plants’ ability to endure external stresses with reproductive success (Freeling 2008; Hanada, Zou et al. Zou et al. 2008; Zhang, Huang et al. Huang et al. 2010a; Filichkin, Priest et al. Priest et al. 2011a; Hu, Lin et al. Lin et al. 2011). While complex gene-gene interactions regulate these responses, they are mediated by biochemical reactions that are part of a plant’s metabolic network (Less and Galili 2008) (Lu, Liu et al. 2009). Genome sequence, gene structure, and functional annotation provide the basis for understanding a genome. However, in order to understand the physiology, development and adaptation of a plant and its interaction with the environment, its metabolic network needs to be deciphered. This network represents a (bio)chemical manifestation of downstream changes in shape, form, growth and development. With this in mind, we developed, curated and annotated RiceCyc, the metabolic pathway network of rice, and is currently hosted in the Gramene database (http://www.gramene.org/pathway/). Through this database we present a snapshot of the substrates, metabolites, enzymes, reactions and pathways of primary and intermediary metabolism in rice. In this report we will provide database insights based on RiceCyc version 3.3. Beside discussing the development of RiceCyc we will demonstrate the capabilities of RiceCyc as a platform for integrating and analyzing multiple omics data sets in terms of knowledge discovery. To emphasize the latter, we present here the first detailed report on full-scale annotation and integrated analysis of the serotonin, auxin IAA (indole-3-acetic acid) and tryptophan biosynthesis pathways in terms of stress and diurnal regulation, using the RiceCyc platform and publicly available rice transcriptomic data sets.

## Results and discussion

### Development of the RiceCyc pathway database

The RiceCyc pathway database currently features 316 metabolic pathways, 2,103 enzymatic reactions, 87 transport reactions and 1,543 compounds and metabolites. 6,643 protein coding genes i.e. about 14% of the total ~47,894 protein coding and tRNA genes that are either known and/or predicted from rice (Yuan, Ouyang et al. 2005) are mapped to these reactions and pathways. This is close to the expected estimate of metabolic gene representation in a proteome, which generally falls in the range of 15-20% of the total protein coding gene set. It may increase depending on the ploidy and ancestral genome duplications in a plant genome, as well as on the quality and depth of the genome’s annotation. For example grape and Eucalyptus genome annotation (Jaiswal et al. unpublished) has revealed that about 30% of the total mapped gene loci are metabolic. Our annotation workflow applied to the BrachyCyc, SorghumCyc and MaizeCyc metabolic networks identified respectively about 30, 32 and 23 percent of the total nuclear encoded protein coding genes as having a potential metabolic role. At the time of building RiceCyc, of the total 47,894 rice genes, we found 29,753 genes to have GO annotations (Ashburner, Ball et al. 2000). These fell into 21,984 biological process, 10,996 cellular component and 27,791 molecular function GO assignments. 7,473 protein coding genes had Enzyme Commission (EC) number annotations and 37,223 had InterPro (Hunter, Apweiler et al. 2009) assignments as part of the first-pass annotations. Furthermore, at the time of building RiceCyc, about 1,400 gene annotations spanning 112 pathways were integrated from the KEGG rice pathways (Masoudi-Nejad, Goto et al. 2007; Kanehisa, Goto et al. Goto et al. 2011). Additional reaction enrichments were introduced by projecting known annotations from MetaCyc (Mueller, Zhang et al. 2003; Zhang, Foerster et al. Foerster et al. 2005; Caspi, Altman et al. Altman et al. 2012), AraCyc (Mueller, Zhang et al. 2003; Zhang, Foerster et al. Foerster et al. 2005) and Plant Metabolic Network (Zhang, Dreher et al. 2010b).

### RiceCyc website user interface

RiceCyc can be accessed through the “Pathways” module of the Gramene database (http://www.gramene.org/pathway/) (Jaiswal 2010; Youens-Clark, Buckler et al. Buckler et al. 2011) where researchers can browse, query, and visualize the data. RiceCyc is hosted together with other monocot plant metabolic pathway databases, namely SorghumCyc for *Sorghum bicolor*, BrachyCyc for *Brachypodium distachyon* and MaizeCyc for *Zea mays* (Monaco, Sen et al. 2013) which are also developed and curated by Gramene. Mirrors of dicot species-specific pathway databases AraCyc (Mueller, Zhang et al. 2003; Zhang, Foerster et al. Foerster et al. 2005), MedicCyc (Urbanczyk-Wochniak and Sumner 2007), PoplarCyc (Zhang, Dreher et al. 2010b), CoffeeCyc and SolCyc (Mueller 2013), and reference databases MetaCyc (Krieger, Zhang et al. 2004; Zhang, Foerster et al. Foerster et al. 2005), PlantCyc (Zhang, Dreher et al. 2010b) and EcoCyc (Latendresse, Paley et al. 2011) are also housed for comparative analysis.

**Figure 1:**
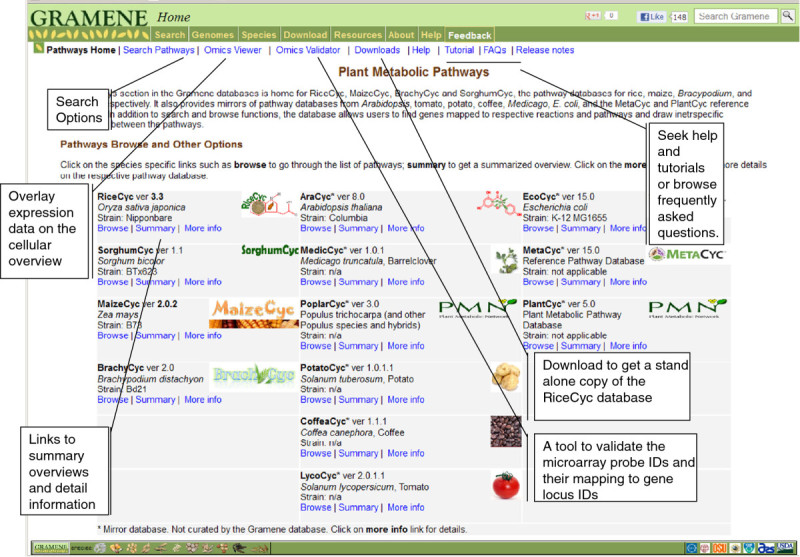
**A screen shot of Gramene’s Pathway module (**http://www.gramene.org/pathway/**).** Various sub menu options include: search pathways, OMICs Viewer, Omics validator, Download, Help, Tutorial, Frequently asked questions (FAQs) and release notes. The pathway database listings included in column-1 the RiceCyc, SorghumCyc, MaizeCyc and BrachyCyc. These are developed by the Gramene database project. Column-2 includes mirror of pathway databases provided by collaborators TAIR, Noble Foundation and SGN. The mirrored databases include the Aracyc (*Arabidopsis*), MedicCyc (Medicago), PoplarCyc (Poplar), PotatoCyc (potato), LycoCyc (tomato) and CoffeaCyc (Coffee). Column-3 includes mirrors of reference pathway databases namely the EcoCyc (E. coli), MetaCyc and PlantCyc.

**Figure 2:**
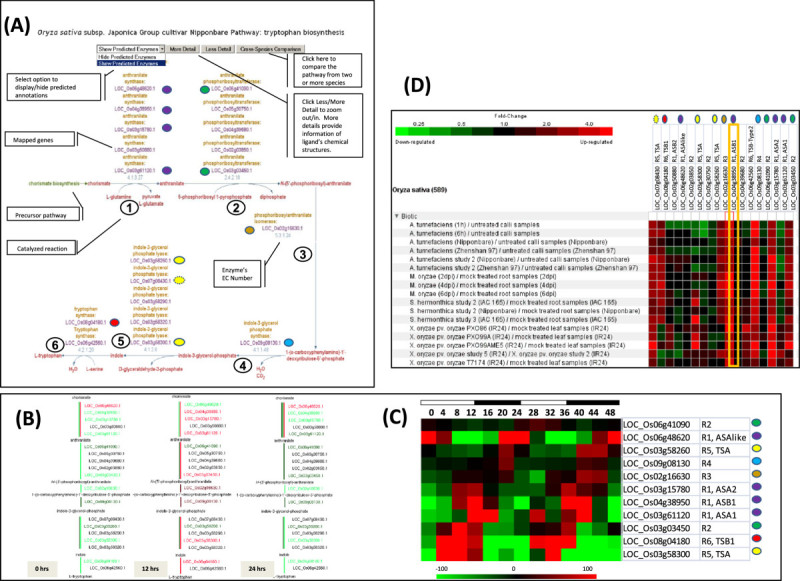
**Gene expression analysis of the genes associated with the enzymes catalyzing the tryptophan biosynthesis.** (**A**) A screen shot of the tryptophan biosynthesis pathway in RiceCyc with details of different objects; precursors on pathways, input and output molecules, enzymes referred by the EC number and proteins/genes associated with the enzymatic activity of a reaction. The pathway pages provide options to display only the experimentally determined protein-enzyme activities and zoom-in (molecular structures of the compounds) and zoom-out (pathway overview) views of pathways and the option to compare similar pathways from other species. The colored dots indicate the cycling genes at each reaction as demarcated in the heatmap in C. The yellow circle with a dotted outline indicates a “cycling’ gene that marginally missed the Q value cutoff (0.79) (**B**) A zoom-out view of the tryptophan biosynthetic pathway with genes overlaid with the expression levels of diurnally regulated transcriptome (green/down-regulated, red/upregulated). The 0, 12 and 24 hrs profiles were chosen to record differences between the expression profiles of the rice homologs mapped to the same enzymatic activity. A majority of genes in the pathway are highly expressed (red colored) at the 12 hr time point (towards the end of the day light cycle) compared to the down regulation in dark (green colored) and at the beginning of the day light cycle. (**C**) A heat map display of the expression pattern of all cycling genes in the pathway with the gene ID, subunit type and reaction of the pathway. Colored circles are used to identify individual reactions. (**D**) Biotic and abiotic stress induction of tryptophan biosynthetic genes based on data compiled in Genevestigator for rice. Expression data available for perturbations were filtered for significance level (p- value <0.05) and fold change (>2, based on gene in column with a yellow outline).

Users can navigate to the desired pathway via ‘search pathways’ or ‘browse’ options (Figure 1), either from an alphabetical list of all pathways or from the hierarchical ontology browser. On pathway detail pages (Figure 2A), graphical diagrams of the pathways can be viewed at different levels of detail. The online comparative analysis tool (http://pathway.gramene.org/comp-genomics) allows users to perform comparisons between any two or more species by selecting their choices and look for common or species-specific pathways, reactions, metabolites etc. Each pathway and reaction details page also provides a cross-species comparison option to compare details of the specific pathway or reaction between any two or more species (Figure 2A).

### Cellular overview, OMICs viewer and omics validator

**Figure 3:**
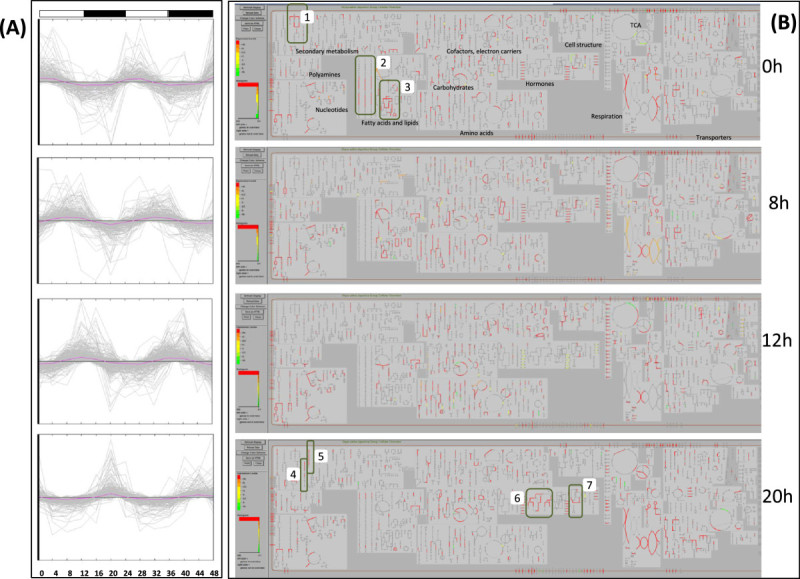
**Expression pattern and metabolic processes of diurnally cycling genes in rice.** (**A**) Expression pattern graphs of genes within the 4 major sub-clusters derived from hierarchical clustering of diurnally cycling genes (Additional file 3: Figure S1A) that mapped to RiceCyc metabolic pathways. (**B**) A metabolic cellular expression overlay of the 4 major diurnal gene expression clusters (the 4 sub-clusters in Additional file 3: Figure S1A and sub-graphs in Additional file 6: Figure S3A above) that show peak expression at 0 h, 8 h, 12 h and 20 h respectively. Boxed pathways: (1) phenylpropanoid (lignin) metabolism, (2) cholesterol biosynthesis, (3) glycolipid and phospholipid desaturation, (4) mevalonate pathway, (5) momilactone pathways, (6) gibberellins biosynthesis, (7) IAA biosynthesis.

The tool Cellular Overview (http://pathway.gramene.org/RICE/expression.html) illustrates the biochemical pathway map within the context of the cell metabolome (Figure 3). The use of the OMICs Viewer in viewing and analyzing gene expression data is illustrated in detail in Figure 3. The OMICs Viewer depends on the Gene ID to map the expression values. Therefore, to help users we have built in an Omics validator tool (http://www.gramene.org/db/omics/validator) for validating and mapping microarray probe IDs, gene names or gene symbols to a locus ID in user provided data files. If the gene IDs are missing in the data file but if there are mappings from the microarray platform specific probe ID to a specific gene, the tool regenerates a valid expression data file for expression data analysis within the OMICs Viewer. The ability to visualize the expression patterns in virtual cell-like simulations and projections on the pathways and reactions helps the user to pinpoint and interpret interesting expression patterns as compared to examining a tabulated text file or annotation and function enrichment data derived from tools such as Blast2GO and BinGO.

### Pathway database curation

Computationally predicted databases suffer both from false negatives and false positives. The primary reference pathway database, MetaCyc that we have used for creating RiceCyc only houses experimentally demonstrated pathways, enzymes, reactions and compounds listed in its reference library which are derived from several organisms including plants. We used taxonomic filtering options to filter out non-plant pathways. This was successful only if the reference library was flagged with the appropriate taxonomic designation. To further reduce the false positives escaping the taxonomic pruning we evaluated the projections against the curated list of plant pathways and reactions provided in the frame format by PlantCyc-PMN (Zhang, Dreher et al. 2010a). In manual curation we regularly update RiceCyc by consulting the latest PlantCyc version and as well as through literature mining. If there are new pathways reported and if corresponding rice orthologs do exist, we create new rice pathways to reduce false negatives. For example, some of the pathways that we curated included those that are only reported in rice, namely, the terpenoid biosynthesis instances of momilactone biosynthesis, Oryzalexin A-F biosynthesis, Oryzalexin S biosynthesis and phytocassane biosynthesis (Shimura, Okada et al. 2007) (Wilderman, Xu et al. 2004) (Xu, Hillwig et al. 2004; Xu, Wilderman et al. Wilderman et al. 2007). These pathways did not get projected with the MetaCyc (v 13.5) prediction and were added through manual curation. However, in the newer MetaCyc version (v 16.0) these pathways have been included in the reference libraries. A case of manual revision involved the cytokinin biosynthesis pathway. Based on published reports, rice gene *LONELYGUY* (*LOG*) (Kurakawa, Ueda et al. 2007) possesses the cytokinin nucleoside 5′-monophosphate phosphoribohydrolase activity and converts cytokinin nucleotides such as trans-zeatin riboside monophosphate (tZPRMP) into isopentenyladenine, a free base form, and ribose 5′-monophosphate and isopentenyladenosine-5′-monophosphate (iPRTP) into isopentenyladenine. These two reactions were novel and were not previously available in the reference MetaCyc and AraCyc databases. A list of pathways that have undergone manual curation involving additions, deletions and revisions is in Additional file 1: Table S1 and is also available from the more information webpage (http://gramene.org/pathway/ricecyc.html) which connects from the ‘Modifications’ hyperlink to the curation webpage (http://gramene.org/pathway/ricecyc_deleted_pathways.html).

### Data availability

The complete RiceCyc database is available online as well as for download from ftp://ftp.gramene.org/pub/gramene/pathways/ricecyc/ in Ocelot (readable by Pathway Tools), BioPAX level-2 and 3, and SBML (Stromback and Lambrix 2005) formats. If desired RiceCyc can be installed for local access on an individual’s desktop or their webserver. Only required items to achieve this are, the RiceCyc database archive from our FTP server (ftp://ftp.gramene.org/pub/gramene/pathways/ricecyc/) and the installation of PathwayTools (available from SRI under their license agreement; http://biocyc.org/download.shtml). The local database setup on desktops running windows, linux, mac or unix environments offer additional operability, such as, faster speed, the opportunity to update the database with proprietary data, and the ability to perform programmatic queries including comparing species wide cellular overviews of pathway networks from multiple species.

### RiceCyc metabolic network for analysis of gene expression

In order to demonstrate the potential impact of RiceCyc on rice research and to test the validity of the network, we mapped existing gene expression data sets to the rice metabolic pathways. By integrating the empirical expression data with the theoretical projections, we wanted to highlight the utility of RiceCyc as a platform for knowledge discovery, through novel interpretation of existing data. As a test case, we used publicly available rice gene expression data sets on stress response and diurnal control of transcription, within the context of the rice metabolic network. We then focused on the effect of these conditions, specifically, on the tryptophan and its derivative auxin and serotonin biosynthetic pathways. In plants, tryptophan is one of the least abundant and biosynthetically energy intensive amino acids. It is also the precursor metabolite for the biosynthesis of plant growth hormone auxin and secondary metabolites phytoalexins, serotonin, glucosinolates and alkaloids, thus playing a critical role in regulating plant development and its response to biotic and abiotic stresses. Specifically, the tryptophan biosynthetic pathway provides precursors for plant defense-related secondary metabolic compounds and is known to be induced by pathogens and elicitors (Radwanski and Last 1995; Zhao and Last 1996; Matsukawa, Ishihara et al. Ishihara et al. 2002; Fujiwara, Maisonneuve et al. Maisonneuve et al. 2012). There is also emerging evidence linking diurnal regulation and immunity in plants. For example, the central clock gene *CCA1* is reported to control genes related to plant defense and growth hormone metabolism in Arabidopsis (Penfield and Hall 2009). We wanted to examine whether these two processes are linked. Therefore, we first examined the diurnal control of gene expression at the genome level and, then, tested whether the biotic stress-induced genes of tryptophan biosynthesis are also diurnally regulated. We further extended our analysis to biosynthetic pathways of tryptophan derivatives serotonin and auxin (IAA).

### Genome scale analysis of gene expression under diurnal control

A large proportion of plant genes show diurnal expression cycling that helps the plant anticipate daily and seasonal changes in the photoperiod and temperature of the growth environment. To illustrate the utility of pathway databases and the flow of data analysis, here we examine a gene expression dataset on diurnal regulation of rice (*O. sativa* ssp. *Japonica*) genes. It was reported (Filichkin, Breton et al. 2011a) that 59% (21,364 out of 35,928 unique gene models) of the transcripts represented on the microarray were cycling under at least one of the several diurnal light and temperature conditions used in the experiment. We analyzed the dataset on rice plants treated with 12 h light/12 h dark photoperiod under constant temperature (LDHH) growth conditions to determine the cycling genes in our metabolic network. Out of the 11,195 genes identified as cycling (Q value cutoff 0.8) under LDHH condition, 2,225 genes mapped to the RiceCyc metabolic network. The gene identifiers and expression values of these genes are listed in Additional file 2: Table S2.

The cluster diagram of gene expression pattern (Additional file 3: Figure S1A) for this gene set shows that there are gene clusters showing cyclic phases of peak expression throughout the day with four major patterns. As observed previously in *Arabidopsis*, poplar and rice (Filichkin, Breton et al. 2011b; Xu, Yang et al. Yang et al. 2011; Michael, Mockler et al. Mockler et al. 2008), a major proportion of genes showed peak expression at light/dark transitions around dawn and dusk. This bimodal distribution is consistent with the expression of many circadian regulated genes in plants. GO enrichment analysis of this gene set (Additional file 3: Figure S1B) shows that diurnally cycling genes span most biosynthetic processes including those of amino acids and derivatives, lipids, carbohydrates and secondary metabolites. Other than the metabolic genes, 260 of the 603 transport genes within the network were found to be under diurnal regulation as well. The plotting of expression values onto the metabolic network using the RiceCyc OMICs Viewer tool confirmed that the cycling genes span most metabolic processes. Figure 3 depicts the OMICs Viewer gene expression views (cellular overview) of the four gene sets (the four sub-clusters) that show first peak expression at 0 h, 8 h, 12 h and 20 h respectively. A majority of metabolic pathways are activated just before or at dusk (8 and 12 h) which include the biosynthesis of amino acids, nucleotides and nucleosides, carbohydrates and cell structure components. Different fatty acid and lipid biosynthetic processes show activation during dawn/0 h and dusk/8 h (e.g. cholesterol, phospholipid, glycolipid). The activation of cell growth and storage related processes at dusk is to be expected and has been reported for starch deposition (Yu, Xu et al. 2011), amino acid metabolism and nucleotide metabolism in rice (Xu, Yang et al. 2011). Secondary metabolism shows a lower proportion of pathways and genes under diurnal control (Figure 3) but phenylpropanoid (lignin) metabolism, mevalonate and momilactone pathways show dawn activation (Figure 3, 0 h and 20 h). Genes mapped to the plant growth hormones gibberellin and auxin (IAA) biosynthesis also showed dawn activation (Figure 3, 0 h and 20 h).

### Diurnal modulation and pathogen induction of tryptophan and derivative biosynthesis

We further examined the gene expression characteristics of the tryptophan biosynthetic pathway and two of its derivatives auxin and serotonin biosynthesis by querying the extensive collection of rice gene expression datasets assembled at Genevestigator (Zimmermann, Hirsch-Hoffmann et al. 2004). As shown in Figure 2A steps 1–6, when gene expression values from diurnal cycling data is mapped on to the tryptophan biosynthetic pathway, it is evident that at least one member of each paralogous set of enzymes/genes from each reaction is under strong diurnal modulation (Figure 2B and 2C). Many of the cycling genes show coordinated regulation with peak expression at the same cycling phase (Figure 2B and 2C, 12 h or dusk). The enzyme anthranilate synthase (EC: 4.1.3.27, reaction 1, Figure 2A) catalyzes a key regulatory reaction of the tryptophan biosynthetic pathway (Li and Last 1996). It is also a heteromeric protein complex composed of anthranilate synthase α (*ASA*) and β (*ASB*) subunits (Poulsen, Bongaerts et al. 1993) (Bohlmann, DeLuca et al. 1995) (Romero, Roberts et al. 1995). Out of the five predicted anthranilate synthase subunits, four exhibit diurnal cycling (Table 1, Figure 2A-C). Of the sub units that are diurnally cycling, three subunits (α subunits *ASA1* and *ASA2* and β subunit *ASB1*) show synchronized cycling, with upregulation around 12 h and 36 h (dusk) . Similarly, the enzyme tryptophan synthase (EC: 4.2.1.20, Step 5 and 6 in Figure 2A) is also a heteromeric protein complex with α and β subunits. Tryptophan synthase-α has 5 predicted subunits and is reported to have indole-3-glycerol phosphate lyase enzymatic activity whereas tryptophan synthase-β with tryptophan synthase activity is composed of two predicted subunits. With reference to diurnal regulation, two tryptophan synthase α subunits and one tryptophan synthase β subunit show diurnal cycling (Table 1).

**Table 1:** **List of rice genes associated with the tryptophan biosynthesis pathway, their EC number assignments, gene nomenclature and the pattern of diurnal expression (Cycling peak)**

Reaction	MSU gene ID	EC number	Enzymatic activity	Gene symbol	Cycling (Peak)
1	LOC_Os06g48620.1	4.1.3.27	anthranilate synthase	*ASA like*	YES (0 h)
1	LOC_Os04g38950.1	4.1.3.27	anthranilate synthase	*ASB1*	YES (12 h)
1	LOC_Os03g15780.1	4.1.3.27	anthranilate synthase	*ASA2*	YES (12 h)
1	LOC_Os03g50880.1	4.1.3.27	anthranilate synthase	*ASB2*	NO
1	LOC_Os03g61120.1	4.1.3.27	anthranilate synthase	*ASA1*	YES (12 h)
2	LOC_Os06g41090.1	2.4.2.18	anthranilate phosphoribosyltransferase	*PAT*	YES (0 h)
2	LOC_Os05g30750.1	2.4.2.18	anthranilate phosphoribosyltransferase	*PAT*	NO
2	LOC_Os04g39680.1	2.4.2.18	anthranilate phosphoribosyltransferase	*PAT*	NO
2	LOC_Os02g03850.1	2.4.2.18	anthranilate phosphoribosyltransferase	*PAT*	NO
2	LOC_Os03g03450.1	2.4.2.18	anthranilate phosphoribosyltransferase	*PAT*	YES (12 h)
3	LOC_Os02g16630.1	5.3.1.24	phosphoribosylanthranilate isomerase	*PAI*	YES (12 h)
4	LOC_Os09g08130.1	4.1.1.48	indole-3-glycerol phosphate synthase	*IGPS*	YES (12 h)
5	LOC_Os03g58260.1	4.1.2.8	indole-3-glycerol phosphate lyase	*TSA*	YES (0 h)
5	LOC_Os07g08430.1	4.1.2.8	indole-3-glycerol phosphate lyase	*TSA*	YES (12 h)
5	LOC_Os03g58290.1	4.1.2.8	indole-3-glycerol phosphate lyase	*TSA*	Not on Array
5	LOC_Os03g58320.1	4.1.2.8	indole-3-glycerol phosphate lyase	*TSA*	Not on Array
5	LOC_Os03g58300.1	4.1.2.8	indole-3-glycerol phosphate lyase	*TSA*	YES (12 h)
6	LOC_Os08g04180.1	4.2.1.20	tryptophan synthase	*TSB1*	YES (12 h)
6	LOC_Os06g42560.1	4.2.1.20	tryptophan synthase	*TSB2 Type-2*	NO

The reaction numbers in column-1 correspond to the reaction numbers listed in Figure 2.

The tryptophan biosynthetic pathway is known for its role in providing precursors for plant defense-related secondary metabolic compounds and the pathway genes are known to be induced by pathogens and elicitors (Radwanski and Last 1995; Zhao and Last 1996; Matsukawa, Ishihara et al. Ishihara et al. 2002). Inspection of Genevestigator expression datasets (Zimmermann, Hennig et al. 2005) for pathogen induction in rice showed that each step of the biosynthetic pathway is broadly induced (Figure 2D) by, 1) bacterial pathogens, *Agrobacterium tumefaciens* and *Xanthomonas oryzae -* causing rice bacterial blight disease, 2) fungal pathogen *Magnaporthe grisea -* causing rice blast disease and 3) by the angiosperm parasitic weed *Striga hermonthica*. The above pathogens induced the genes *ASB1* and *ASA2*, *PAT* (LOC_Os03g03450), *PAI, IGPS, TSA* (LOC_Os07g08430) and *TSB1*. Together these genes cover all six reaction steps in the tryptophan biosynthesis pathway. Interestingly, all the above genes/enzymes also exhibit synchronized diurnal cycling as described previously (Figure 2B and 2C). Induction of the full tryptophan biosynthetic pathway by taxonomically diverse pathogens and diurnal regulation of the same suite of genes is a novel finding and was facilitated by the development of RiceCyc. This discovery supports the emerging evidence from Arabidopsis linking circadian clock and immunity in plants (Wang, Barnaby et al. 2011), where the Arabidopsis central clock gene *CCA1* is reported to control genes related to plant defense and growth hormone metabolism (Penfield and Hall 2009). It is noteworthy that some genes that don’t show broad spectrum pathogen response induction may show response patterns preferential to specific pathogens, e.g. *TSA* (LOC_Os03g58300.1) shows preferred induction after *Striga* and *Xanthomonas* treatments whereas *ASB2, ASA1*, and *TSB Type-2* show preferred induction after *Xanthomonas* treatment.

While the multisubunit enzymes anthralinate and tryptophan synthases within the tryptophan biosynthesis pathway are shown to be pathogen-induced, there is also evidence for differential regulation of specific anthranilate synthase subunits under biotic and abiotic stress in both rice and Arabidopsis (Ishihara, Hashimoto et al. 2008a) (Kanno, Kasai et al. 2004). Specifically, anthranilate synthase α-subunit (*ASA1*) is reported not to be induced by pathogens or elicitors (Ishihara, Hashimoto et al. 2008b) (Kanno, Kasai et al. 2004) and its *ASA1-ASB1* heteromer is reported to be less sensitive to feedback inhibition by tryptophan when compared to the alternative heterodimer formed by *ASA2-ASB1*(Kanno et al., 2004). This was corroborated in our observations where *ASA1* was only slightly induced by *Xanthomonas*, whereas *ASA2* and *ASB1* showed highly inducible expression in response to pathogens or elicitors. Interestingly we also found *ASA2* and *ASB1* expression to be diurnally synchronized, implying an overlap between gene isoforms which are pathogen-induced and diurnally modulated, thus suggesting their importance as key regulators of metabolic flux within the tryptophan biosynthetic pathway. When we queried the rice functional gene network RiceNet (described later: Lee, Seo et al. 2011), we were intrigued to find that both *ASB1* and *ASA2* interact with the protein *XA21* (LOC_Os11g36180). *XA21* is a pattern recognition receptor kinase that mediates resistance to the Gram-negative bacterium *Xanthomonas oryzae* pv. oryzae (Song, Wang et al. 1995; Wang, Song et al. Song et al. 1996; Yoshimura, Yamanouchi et al. Yamanouchi et al. 1998). The previously reported results from tryptophan feedback inhibition and the current observations on *Xanthomonas* induction of *ASB1* and its interaction with *XA21* lead us to propose that *ASA2-ASB1* may play a key role in connecting the *Xanthomonas*-induced defense signaling network and the tryptophan derivative metabolic network in rice.

Tryptophan synthase α-subunit (*TSA*) having the indole-3-glycerol phosphate lyase activity and tryptophan synthase β-subunit (*TSB*) are known to be induced by biotic and abiotic stresses in Arabidopsis (Zhao and Last 1996). In the current study, RiceCyc recognizes five paralogs encoding *TSA* and two encoding *TSB* (Figure 2A). Of these, the *TSA* encoded by LOC_Os07g08430 is induced by multiple pathogens whereas the *TSA* encoded by LOC_Os03g58300 is preferentially induced by the pathogen *Xanthomonas oryzae* and the parasite *Striga*. The *TSA* LOC_Os03g58260 is not induced by pathogens. The remaining two TSA genes are not represented in the publicly available array datasets that we have analyzed. Similarly, one of the tryptophan synthase β subunits, *TSB1* is induced by a broad set of pathogens with the exception of *X. oryzae*. The tryptophan synthase β subunit *TSB2*, which is a ‘type-2’ (Xie, Forst et al. 2002) *TSB* is preferentially induced by *X. oryzae*. The ‘type 2’ TS-β subunits are present in all sequenced genomes. In Arabidopsis and maize, they are expressed in multiple tissues and are enzymatically very active in converting indole to tryptophan. However, since no Arabidopsis mutants have been reported, no specific role has been designated and has been suggested to be functionally redundant (Yun et al. 2010). On the contrary, the observation of preferential induction of a ‘type-2’ *TSB* (*TSB2*) by *X. oryzae* in the current study suggests a specific role for the ‘type 2’ *TSB*, at least in rice.

An intriguing pattern emerged during the integration of multiple gene expression data sets on pathogen induction with the diurnal control study. As seen in Figure 2C and 2D, gene isoforms within tryptophan biosynthesis which are induced by a broad set of pathogens are also diurnally controlled, synchronized and show peak expression at 12 hrs; gene isoforms which are induced specifically or mostly by *X. oryzae* may or may not be diurnally modulated.

### Serotonin biosynthesis

**Figure 4:**
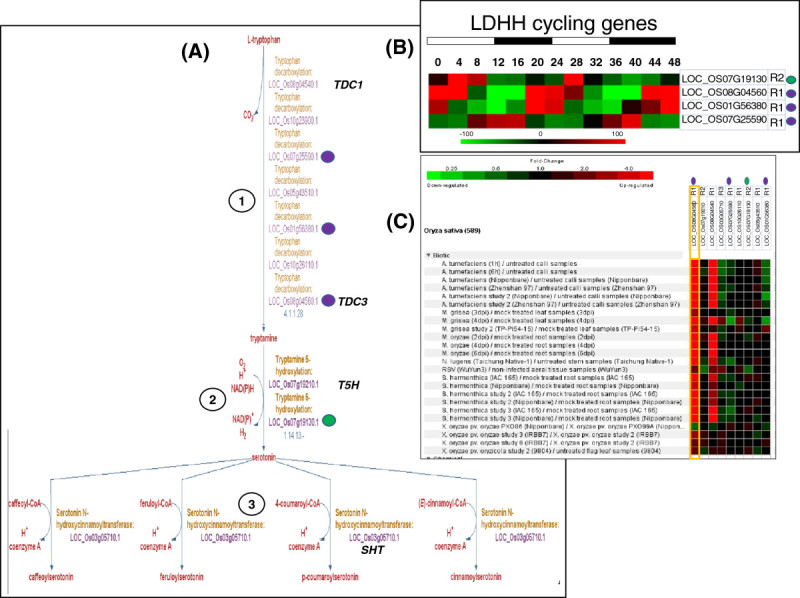
**Gene expression analysis of the genes associated with enzymes catalyzing serotonin biosynthesis.** (**A**) A zoom-in view of the serotonin biosynthetic pathway. (**B**) Heat map of expression pattern of all cycling genes in the pathway with the gene ID and reaction of the pathway. Colored circles are used to identify individual reactions. (**C**) Biotic and abiotic stress induction of tryptophan biosynthetic genes based on data compiled in Genevestigator for rice. Expression data available for perturbations were filtered for significance level (p- value <0.05) and fold change (>2, based on gene in column with a yellow outline).

Serotonin (5-hydroxytryptamine), a neurotransmitter is well known to play several key physiological and behavior-associated roles in animals. Serotonin, its precursor molecules and interactors such as their receptors and transport proteins, are also known to show diurnal modulation in animals. Interestingly the serotonin molecule is also known to be present in a wide range of plant species (Roshchina 2001) and is thought to have occurred in plants long before the evolution of animals (Azmitia 2001). Multiple roles have been reported or proposed for serotonin in plants including flower and seed development (Murch, Alan et al. 2009); (Murch and Saxena 2002), senescence (Kang, Kim et al. 2009; Park, Lee et al. Lee et al. 2012), defense responses (Ishihara, Hashimoto et al. 2008a; Ishihara, Hashimoto et al. Hashimoto et al. 2008a; Ishihara, Nakao et al. Nakao et al. 2011) and light mediated co-regulation of melatonin biosynthesis (Byeon, Park et al. 2012). The amino acid L-tryptophan is the precursor of serotonin. L-tryptophan is decarboxylated by tryptophan decarboxylase (TDC) to tryptamine and then hydroxylated by tryptamine 5-hydroylase to serotonin (Figure 4A) (Park, Kang et al. 2011). TDC has been described to function as a homodimer and the decarboxylation reaction it catalyzes is a known rate-limiting reaction of the serotonin biosynthesis pathway (Park, Kang et al. 2009). There are 7 TDC-like enzymes identified in RiceCyc and two of them, encoded by LOC_Os08g04540-*TDC1* and LOC_Os08g04560-*TDC3*, are highly induced by both abiotic and biotic stresses (Figure 4C). Both *TDC1* and *TDC* 3 are induced by a broad spectrum of pathogens. However, when compared to *TDC1, TDC3* is significantly induced by *X. oryzae*. When we analyzed the diurnal patterns of these genes, we found *TDC3* to be under diurnal modulation with upregulation at 0–4, 20–24, and 44–48 hour time points (Figure 4B and C). It is noteworthy that this diurnal regulation has some degree of overlap with the diurnal modulation of *TSA* and *TSB1* genes of the tryptophan biosynthesis pathway (see Figures 2C and 3B). As described above *TDC* is highly induced by pathogens whereas tryptamine 5-hydroxylases and serotonin N-hydroxycinnamoyltransferase are not significantly induced by pathogens (Figure 4C). *TDC* therefore could be considered as the rate-limiting reaction of the serotonin biosynthesis pathway which is consistent with several previous studies (Kang, Kim et al. 2009); (Ishihara, Hashimoto et al. 2008b); (Kanjanaphachoat, Wei et al. 2012).

### Auxin (IAA) biosynthesis

*De novo* synthesis of IAA in plants is complex and can occur via tryptophan (Trp)-dependent or Trp-independent pathways. To date, a complete *de novo* auxin biosynthetic pathway has not been well-defined for plants (Zhao 2010; Mashiguchi, Tanaka et al. Tanaka et al. 2011). The genetic and biochemical studies indicate amino acid tryptophan as the main precursor of IAA biosynthesis in plants (Cohen, Slovin et al. 2003; Zhao 2010; Mashiguchi, Tanaka et al. Tanaka et al. 2011; Zhao 2012). Therefore, we examined the expression characteristics of the predicted Trp-dependent pathways for rice. The gene expression analysis revealed that all reactions with assigned genes (except the tryptophan monooxygenase, reaction-7 in Additional file 4: Figure S2A) show gene members with strong diurnal expression patterns (Additional file 4: Figure S2B).

Analysis of the various publicly available stress-treated and developmental phase rice gene expression datasets suggest that unlike the tryptophan biosynthetic pathway (Figure 2D), auxin biosynthesis genes do not show widespread induction under biotic stresses (Additional file 4: Figure S2C). However, during the developmental stages of germination, all reactions, excluding the Indole-3-acetonitrile nitrilase (Additional file 4: Figure S2A reaction-9) catalyzed reaction (Additional file 4: Figure S2C, ‘Other’-aerobic germination study) show induced gene expression. General pathogen induction was only observed for 2 tryptophan decarboxylases, LOC_Os08g04560 and LOC_Os08g04540 with the former also showing induction during germination (Additional file 4: Figure S2C). This reaction is shared with the serotonin biosynthetic pathway (reaction-1, Figure 4A) described in the preceding section. IAA biosynthesis via the indole-pyruvic acid (IPA) pathway has garnered more experimental support since the characterization of *Arabidopsis tryptophan amino transferase 1* (*TAA1*) gene mutants defective in the conversion of L-tryptophan to IPA (Stepanova, Robertson-Hoyt et al. 2008; Tao, Ferrer et al. Ferrer et al. 2008). Although the subsequent reaction of the IPA derived IAA biosynthetic pathway involving the conversion of IPA to indole acetaldehyde by indole-3-pyruvate decarboxylase (EC:4.1.1.74, reaction-2 in Additional file 4: Figure S2A) is well characterized in microbes, it is yet to be characterized in *Arabidopsis* and other plants (Won, Shen et al. Won et al. 2011). Five proteins have been predicted for the catalysis of this reaction in RiceCyc (Additional file 4: Figure S2A) and interestingly, two of these, LOC_Os01g06660 and LOC_Os05g39310 show upregulation during germination (Additional file 4: Figure S2C). These two rice genes and homologous *Arabidopsis* pyruvate decarboxylase genes AT4G33070, AT5G01320, AT5G01330 and AT5G54960 are not well characterized in AraCyc, but they do show homology to bacterial enzymes that carry out this reaction efficiently (Schutz, Golbik et al. 2003). Therefore, the two rice and 4 *Arabidopsis* homologs are good candidates for further investigation delineating this undefined reaction of IAA biosynthesis via IPA in plants. It has been suggested that in *Arabidopsis*, the YUCCA family of monooxygenases convert IPA to IAA (IPA route) in contrast to the currently described YUC route of IAA synthesis (Mashiguchi, Tanaka et al. 2011; Zhao 2012). This new 2-step route, tryptophan-IPA-IAA, is not currently indicated in RiceCyc but could in fact be functional in rice, as TAA (LOC_Os01g07500) and YUC (LOC_Os12g32750, LOC_Os04g03980) genes are induced during germination and they also show tissue specific expression patterns that are consistent with established auxin biosynthetic sites such as root tip/radical and stele (Additional file 5: Figure S4C).

### Discovering interconnections using the rice functional gene network (RiceNet) and RiceCyc

To examine the regulation of tryptophan biosynthesis at the protein interaction level, we looked at regulatory proteins that interact with the tryptophan biosynthetic pathway genes. To do so we probed the rice probabilistic functional network RiceNet (Lee, Seo et al. 2011) with the list of 19 enzyme coding genes associated with the tryptophan biosynthesis pathway in rice. The query resulted in finding 1,195 interactions involving 537 genes including the 19 tryptophan biosynthesis pathway genes and 518 other genes (Additional file 6: Figure S3, Additional file 7: Table S3). Examining this list of 518 genes for overrepresentation of Gene Ontology (Ashburner, Ball et al. 2000) categories revealed enrichment in regulatory processes related to protein-DNA complex assembly, ubiquitin-dependent protein catabolism, kinase/MAP kinase activity and transporter activity. This is consistent with the existing knowledge in other plant systems, for example, MAPK cascades (MPK3/MPK6) are known to regulate biosynthesis of tryptophan and its derivatives phytoalexin and camalexin in Arabidopsis (Xu et al. 2007), and ubiquitination regulates many aspects of stress response including activation of stress response pathways and recycling damaged proteins (Flick and Kaiser 2012).

In addition to regulatory processes, this list of 518 genes was also enriched for processes related to sulfur amino acid biosynthesis (e.g. cysteine and serine). These amino acids are reported to provide a protective role during induced oxidative stress and play a critical role in the initiation of hypersensitive response and immunity in Arabidopsis (Alvarez, Bermudez et al. 2012). The interactions observed for genes involved in tryptophan, cysteine and serine biosynthesis suggest commonality in stress-induced regulation of these pathways encompassing a community of gene loci acting together as a subnetwork. Five genes of this list, namely LOC_Os06g49430 (*OsMPK12*), LOC_Os01g47530 (*OsMPK8*), LOC_Os03g57220 (*GIP1*), LOC_Os05g07860 (*SAB10*) and LOC_Os11g36180 (*XA21*) were also components of the “100 protein interactors” that were reported to be the key regulators within the rice biotic and abiotic stress response network that have been identified through Y2H screening (Seo, Chern et al. 2011). *XA21* is a pattern recognition receptor kinase that mediates resistance to the Gram-negative bacterium *Xanthomonas oryzae* pv. oryzae (Xoo) through activation of defense response genes in the nucleus (Song, Wang et al. 1995; Wang, Song et al. Song et al. 1996; Yoshimura, Yamanouchi et al. Yamanouchi et al. 1998). We observed that *XA21* (LOC_Os11g36180) interacts with anthranilate synthase-β subunits *ASB1* and *ASB2* that are components of the key regulatory reaction of the tryptophan biosynthetic pathway. In addition, *SAB10* (LOC_Os05g07860), a component of the ethylene mediated abiotic stress response subnetwork in rice (Seo, Chern et al. 2011), also interacts with the β-subunits of anthranilate synthase (similar to *XA21*) and both α subunits of this key regulatory enzyme complex. Many of these interactions based on co-induction and expression were reported in rice and Arabidopsis and are consistent with our analysis (Li and Last 1996; Cheong, Chang et al. Chang et al. 2002; Kanno, Kasai et al. Kasai et al. 2004; Kim, Lee et al. Lee et al. 2005; Matsuda, Yamada et al. Yamada et al. 2005; Hong, Peebles et al. Peebles et al. 2006; Wakasa, Hasegawa et al. Hasegawa et al. 2006; Dubouzet, Ishihara et al. Ishihara et al. 2007; Matsuda, Wakasa et al. Wakasa et al. 2007; Ishihara, Hashimoto et al. Hashimoto et al. 2008a).

## Conclusions

We have developed a metabolic network for rice with mappings of annotated genes to individual reactions, and we have discussed how it serves as a platform to visualize and analyze genome scale datasets and carryout comparative analysis. The integrated bioinformatic analyses of publicly available datasets revealed the coordinated regulation of gene family members in the tryptophan biosynthetic pathway and its downstream serotonin and IAA biosynthesis pathways. Specific gene family members along with most reactions of these pathways are highly and specifically induced when rice plants are under various biotic and abiotic stresses and support the notion that the regulation of tryptophan biosynthesis responds to the needs of secondary metabolites and derivatives in rice plants more so than to the needs of protein synthesis. The tissue specific expression pattern shows induction of tryptophan biosynthetic genes in root tip and stele (Additional file 5: Figure S4), which are also the active sites of downstream pathways for auxin and serotonin biosynthesis. Several lines of evidence in rice, Arabidopsis and other plants, along with this *in-silico* study, support the hypothesis that serotonin, auxin and tryptophan biosynthetic pathways are under diurnal control for expression and possibly have a role in root system development and response to biotic and abiotic stress. A recent report identified the rice *Sekiguchi lesion* (*SL*) gene, which encodes a cytochrome P450 monooxygenase that bears tryptamine 5-hydroxylase activity. It catalyzes the conversion of tryptamine to serotonin. This is a plant-specific reaction that is not found in humans (Fujiwara, Maisonneuve et al. 2012). According to Fujiwara *et al.* (Fujiwara, Maisonneuve et al. 2012), expression of *SL* was induced by chitin elicitor or by treating rice plants with *Magnaporthe grisea*, the causal agent for rice blast disease. The exogenous application of serotonin was found to induce expression of defense related genes thereby enhancing resistance. A similar application of serotonin in rice suspension cultures induced cell death. Together with several earlier reports and analysis of publicly available rice gene expression and gene-gene interaction datasets, RiceCyc enabled the identification of key enzymes and genes that are regulated and critical for pathogen response in the pathways examined. This information is valuable for the design of hypothesis-driven future experiments on the metabolic engineering of plants. The co-examination of diurnal and pathogen response activation showed strong evidence of a link between circadian control and activation of core tryptophan pathway and derivative serotonin biosynthesis genes under pathogen treatment. Though the analysis shown in this communication was designed to build a hypothesis-driven analysis of publicly available biological datasets, much remains to be confirmed by carrying out in-depth experiments on the findings of this report.

## Methods

### Gene annotations

The rice protein sequences were downloaded from Gramene’s Rice Ensembl genome. The proteins were submitted to an annotation workflow that included HMM-based annotations from InterPro, SMART, PRINT, Prosite and Pfam. The second round of annotations involved cellular localization prediction based on Predotar for plastid and mitochondria (Small, Peeters et al. 2004), SignalP for plastid mitochondria and secretory pathways (Emanuelsson et al. 2007) and TMHMM for transmembrane (Sonnhammer, von Heijne et al. 1998) location. The localization results were stringently filtered for quality acceptance. This included acceptance of any score above 0.5 as a predicted location, with a second filter suggesting that any score =/> 0.75 received strong confidence and those with =/< 0.5-0.74 received less confidence. After these predictions were compiled, several ontology mapping files (e.g. InterPro2go, ec2go, prosite2go, pfam2go, metacyc2go) provided by GO (Ashburner, Ball et al. 2000) were used to create the GO assignments for each gene product (Yamazaki and Jaiswal 2005). Annotations were further enriched by importing the *Arabidopsis* gene ontology and biochemical pathway (AraCyc) annotations to the rice gene products based on gene-gene homology evaluated by Ensembl Compara datasets. The final step was curation and quality check, where a human expert would review and accept, modify or reject the computational predictions. The rice genes used for the current build are based on MSU6 rice genome annotations. However, in the future we will be updating them with RAP (Rice Annotation Project) identifiers and upcoming revised IRGSP (International Rice Genome Sequencing Project) annotations for *Oryza sativa* ssp. *japonica* cultivar Nipponbare.

### Predicting metabolic pathway network

After compiling all gene annotations, an input file with the desired file format was prepared for the PathoLogic prediction option provided by Ptools software v13.5 (Karp, Paley et al. 2002) to project the rice metabolic pathways. The MetaCyc-based pathway prediction, species filtering and enrichment of plant specific pathways using PlantCyc v3.0 were essentially similar to methods adopted for building MaizeCyc (Monaco, Sen et al. 2013). The reference pathways listed in MetaCyc v13.5 included 1600 species and PlantCyc v3.0 available from Plant Metabolic Network (PMN) project (Zhang, Dreher et al. 2010a) included approximately 300 species of pathway data as reference. The initial build was further subjected to (1) mapping annotations against the Uniprot features and annotations, (2) picking up annotations from KEGG that were missed by our pipeline, (3) second-level pruning for species filters, (4) manual quality control and (5) manual curation of new and existing pathways to integrate rice specific data,and literature citations.

### Gene expression data analysis

The analysis of diurnal gene expression was conducted as described in Filichkin, Breton et al. (2011b). The datasets can be downloaded from the diurnal project website (http://diurnal.mocklerlab.org/). Functional enrichment analysis using GO category over-representation was carried out using the network visualization program Cytoscape (Killcoyne, Carter et al. 2009) with the BiNGO plugin (Maere, Heymans et al. 2005). BinGO looks at the enrichment of Gene Ontology assignments in a given list of genes. For determination of over- representation, the Benjamini and Hochberg FDR-adjusted significance level cutoff was 0.05 (Hochberg and Benjamini 1990). The color intensity depicted on circles are based on overrepresentation significance level (yellow = FDR below 0.05) while the radius of each circle indicated the number of genes in each category. Based on the over-represented gene list and their mapping to known and predicted pathways, the test cases of tryptophan, auxin and serotonin biosynthesis pathways were specifically selected for their known association in plant development and response to biotic and abiotic stresses. Genevestigator (Grennan 2006), a widely used resource for investigating spatio-temporal and response patterns of genes was used to determine expression characteristics of gene sets assigned to specific pathways. At the time of analysis it housed 80 different experiments on rice with 1677 samples and 146 conditions that were normalized across all samples. We probed the gene sets for tissue type and perturbation-induced gene expression levels. To filter the perturbation datasets, the p-value was set at <0.05 and the fold change was set at >2 for one of the genes showing the highest up-regulation (boxed with a yellow outline in Figures 2D, 4C and Additional file 4: Figure S2C). To find proteins that interact with tryptophan biosynthetic pathway enzymes we probed the rice probabilistic functional network RiceNet (http://www.functionalnet.org/ricenet/). It is primarily a genome-scale network based on predicted protein-protein interactions and co-expression.

### Online pathway tools and comparative species pathways

After building the RiceCyc pathway database, it was hosted on a webserver for online use, following installation guidelines provided by the PTools software (Karp, Paley et al. 2002). The species-specific pathway databases and reference pathway databases were downloaded from their respective online sources and implemented for online comparative analysis.

## Electronic supplementary material

Additional file 1: Table S1: List of pathways that have undergone manual curation. (XLSX 12 KB)

Additional file 2: Table S2: Gene expression values of diurnally cycling rice genes mapped to RiceCyc pathways. The headers of data columns indicate the diurnal sampling time point starting with the beginning of the light period (0 h). (XLSX 379 KB)

Additional file 3: Figure S1: Expression pattern and over-represented metabolic processes of diurnally cycling genes. (A) Hierarchical clustering of gene expression pattern of diurnally cycling genes in rice, mapping to RiceCyc metabolic pathways. Mean centered expression levels are represented with green denoting downregulation, and red indicating upregulation. (B) Over-represented gene ontology categories of gene set represented in (A). (PPTX 505 KB)

Additional file 4: Figure S2: Gene expression analysis of the genes associated with enzymes catalyzing IAA biosynthesis. (A) A zoom-in view of IAA biosynthetic pathway. (B) Heat map of expression patterns of all cycling genes in the pathway with the gene ID and reaction of the pathway. Colored circles are used to identify individual reactions. (C) Biotic and abiotic stress induction of tryptophan biosynthetic genes based on data compiled in Genevestigator for rice. Expression data available for perturbations were filtered for significance level (p- value <0.05) and fold change (>2, based on gene in column with a yellow outline). (PPTX 221 KB)

Additional file 5: Figure S4: Tissue type expression pattern of genes in tryptophan biosynthesis (A), serotonin biosynthesis (B), and IAA biosynthesis (C) from data compiled in Genevestigator. (PPTX 178 KB)

Additional file 6: Figure S3: Rice tryptophan biosynthetic enzyme interactome based on RiceNet. Blue circles on the periphery of the interactome represent tryptophan biosynthetic enzymes. Green circles represent interacting proteins identified from RiceNet. Three of the five anthranilate phosphoribosyltransferase paralogs (LOC_Os06g41090, LOC_Os04g39680, LOC_Os05g30750) did not share interactions with other tryptophan biosynthetic enzymes and clustered separately (lower left corner of Figure). (PPTX 2 MB)

Additional file 7: Table S3: Rice tryptophan biosynthetic enzyme interactors based on the RiceNet. We probed the rice probabilistic functional network, RiceNet (http://www.functionalnet.org/ricenet/) to find proteins that interact with tryptophan biosynthetic pathway enzymes. Nodes represent genes/proteins while edges represent interactions. Column-A is node/interactor-1, column-B is relationship (network edge) and Column-C is node/interactor-2. (XLSX 33 KB)

Below are the links to the authors’ original submitted files for images.

Authors’ original file for figure 1

Authors’ original file for figure 2

Authors’ original file for figure 3

Authors’ original file for figure 4

Authors’ original file for figure 5

Authors’ original file for figure 6

## References

[CR1] Alvarez C, Bermudez MA (2012). Cysteine homeostasis plays an essential role in plant immunity. New Phytol.

[CR2] Ashburner M, Ball CA (2000). Gene ontology: tool for the unification of biology. The Gene Ontology Consortium. Nat Genet.

[CR3] Azmitia EC (2001). Modern views on an ancient chemical: serotonin effects on cell proliferation, maturation, and apoptosis. Brain Res Bull.

[CR4] Bohlmann J, DeLuca V (1995). Purification and cDNA cloning of anthranilate synthase from Ruta graveolens: modes of expression and properties of native and recombinant enzymes. Plant J.

[CR5] Byeon Y, Park S (2012). Light-regulated melatonin biosynthesis in rice during the senescence process in detached leaves. J Pineal Res.

[CR6] Caspi R, Altman T (2012). The MetaCyc database of metabolic pathways and enzymes and the BioCyc collection of pathway/genome databases. Nucleic Acids Res.

[CR7] Cheong YH, Chang HS (2002). Transcriptional profiling reveals novel interactions between wounding, pathogen, abiotic stress, and hormonal responses in Arabidopsis. Plant Physiol.

[CR8] Childs KL, Davidson RM (2011). Gene coexpression network analysis as a source of functional annotation for rice genes. PLoS One.

[CR9] Cohen JD, Slovin JP (2003). Two genetically discrete pathways convert tryptophan to auxin: more redundancy in auxin biosynthesis. Trends Plant Sci.

[CR10] Dal'Molin CG, Quek LE (2010). C4GEM, a genome-scale metabolic model to study C4 plant metabolism. Plant Physiol.

[CR11] Dubouzet JG, Ishihara A (2007). Integrated metabolomic and transcriptomic analyses of high-tryptophan rice expressing a mutant anthranilate synthase alpha subunit. J Exp Bot.

[CR12] Emanuelsson O, Brunak S (2007). Locating proteins in the cell using TargetP, SignalP and related tools. Nat Protoc.

[CR13] Ficklin SP, Feltus FA (2011). Gene coexpression network alignment and conservation of gene modules between two grass species: maize and rice. Plant Physiol.

[CR14] Filichkin SA, Breton G (2011). Global profiling of rice and poplar transcriptomes highlights key conserved circadian-controlled pathways and cis-regulatory modules. PLoS One.

[CR15] Filichkin SA, Priest HD (2011b). Genome-wide mapping of alternative splicing in Arabidopsis thaliana. Genome Res.

[CR16] Flick K, Kaiser P (2012). Protein degradation and the stress response. Semin Cell Dev Biol.

[CR17] Freeling M (2008). The evolutionary position of subfunctionalization, downgraded. Genome Dyn.

[CR18] Fujiwara T, Maisonneuve S (2012). Sekiguchi lesion gene encodes a cytochrome P450 monooxygenase that catalyzes conversion of tryptamine to serotonin in rice. J Biol Chem.

[CR19] Grennan AK (2006). Genevestigator. Facilitating web-based gene-expression analysis. Plant Physiol.

[CR20] Hanada K, Zou C (2008). Importance of lineage-specific expansion of plant tandem duplicates in the adaptive response to environmental stimuli. Plant Physiol.

[CR21] Hochberg Y, Benjamini Y (1990). More powerful procedures for multiple significance testing. Stat Med.

[CR22] Hong SB, Peebles CA (2006). Expression of the Arabidopsis feedback-insensitive anthranilate synthase holoenzyme and tryptophan decarboxylase genes in Catharanthus roseus hairy roots. J Biotechnol.

[CR23] Hu C, Lin SY (2011). Recent gene duplication and subfunctionalization produced a mitochondrial GrpE, the nucleotide exchange factor of the Hsp70 complex, specialized in thermotolerance to chronic heat stress in Arabidopsis. Plant Physiol.

[CR24] Hunter S, Apweiler R (2009). "InterPro: the integrative protein signature database". Nucleic Acids Res.

[CR25] Hunter S, Jones P (2012). "InterPro in 2011: new developments in the family and domain prediction database". Nucleic Acids Res.

[CR26] Ishihara A, Hashimoto Y (2008). Induction of serotonin accumulation by feeding of rice striped stem borer in rice leaves. Plant Signal Behav.

[CR27] Ishihara A, Hashimoto Y (2008). The tryptophan pathway is involved in the defense responses of rice against pathogenic infection via serotonin production. Plant J.

[CR28] Ishihara A, Nakao T (2011). Probing the role of tryptophan-derived secondary metabolism in defense responses against Bipolaris oryzae infection in rice leaves by a suicide substrate of tryptophan decarboxylase. Phytochemistry.

[CR29] Jaiswal P (2010). Gramene database: a hub for comparative plant genomics. Methods Mol Biol.

[CR30] Kanehisa M, Goto S (2011). "KEGG for integration and interpretation of large-scale molecular data sets.". Nucleic Acids Res.

[CR31] Kang K, Kim YS (2009). Senescence-induced serotonin biosynthesis and its role in delaying senescence in rice leaves. Plant Physiol.

[CR32] Kanjanaphachoat P, Wei BY (2012). "Serotonin accumulation in transgenic rice by over-expressing tryptophan decarboxlyase results in a dark brown phenotype and stunted growth". Plant Mol Biol.

[CR33] Kanno T, Kasai K (2004). In vitro reconstitution of rice anthranilate synthase: distinct functional properties of the alpha subunits OASA1 and OASA2. Plant Mol Biol.

[CR34] Karp PD, Paley S (2002). The Pathway Tools software. Bioinformatics.

[CR35] Killcoyne S, Carter GW (2009). Cytoscape: a community-based framework for network modeling. Methods Mol Biol.

[CR36] Kim DS, Lee IS (2005). Characterization of the altered anthranilate synthase in 5-methyltryptophan-resistant rice mutants. Plant Cell Rep.

[CR37] Krieger CJ, Zhang P (2004). "MetaCyc: a multiorganism database of metabolic pathways and enzymes". Nucleic Acids Res.

[CR38] Kurakawa T, Ueda N (2007). Direct control of shoot meristem activity by a cytokinin-activating enzyme. Nature.

[CR39] Kuroha T, Tokunaga H (2009). Functional analyses of LONELY GUY cytokinin-activating enzymes reveal the importance of the direct activation pathway in Arabidopsis. Plant Cell.

[CR40] Latendresse M, Paley S (2011). Browsing metabolic and regulatory networks with BioCyc. Methods Mol Biol.

[CR41] Lee I, Seo YS (2011). Genetic dissection of the biotic stress response using a genome-scale gene network for rice. Proc Natl Acad Sci USA.

[CR42] Less H, Galili G (2008). Principal transcriptional programs regulating plant amino acid metabolism in response to abiotic stresses. Plant Physiol.

[CR43] Li J, Last RL (1996). The Arabidopsis thaliana trp5 mutant has a feedback-resistant anthranilate synthase and elevated soluble tryptophan. Plant Physiol.

[CR44] Lu SX, Liu H (2009). A role for protein kinase casein kinase2 alpha-subunits in the Arabidopsis circadian clock. Plant Physiol.

[CR45] Maere S, Heymans K (2005). BiNGO: a Cytoscape plugin to assess overrepresentation of gene ontology categories in biological networks. Bioinformatics.

[CR46] Mashiguchi K, Tanaka K (2011). The main auxin biosynthesis pathway in Arabidopsis. Proc Natl Acad Sci USA.

[CR47] Masoudi-Nejad A, Goto S (2007). EGENES: transcriptome-based plant database of genes with metabolic pathway information and expressed sequence tag indices in KEGG. Plant Physiol.

[CR48] Matsuda F, Wakasa K (2007). Metabolic flux analysis in plants using dynamic labeling technique: application to tryptophan biosynthesis in cultured rice cells. Phytochemistry.

[CR49] Matsuda F, Yamada T (2005). Characterization of tryptophan-overproducing potato transgenic for a mutant rice anthranilate synthase alpha-subunit gene (OASA1D). Planta.

[CR50] Matsukawa T, Ishihara A (2002). Induction of anthranilate synthase activity by elicitors in oats. Z Naturforsch C.

[CR51] Michael TP, Mockler TC (2008). Network discovery pipeline elucidates conserved time-of-day-specific cis-regulatory modules. PLoS Genet.

[CR52] Monaco M, Sen T (2013). Maize Metabolic Network Construction and Transcriptome Analysis. The Plant Genome.

[CR53] Monaco M, Sen T (2012). Maize Metabolic Network Construction and Transcriptome Analysis. The Plant Genome.

[CR54] Mueller L (2013). SolCyc Solanaceae Pathway databases.

[CR55] Mueller LA, Zhang P (2003). AraCyc: a biochemical pathway database for Arabidopsis. Plant Physiol.

[CR56] Murch SJ, Alan AR (2009). Melatonin and serotonin in flowers and fruits of Datura metel L. J Pineal Res.

[CR57] Murch SJ, Saxena PK (2002). Mammalian neurohormones: potential significance in reproductive physiology of St. John's wort (Hypericum perforatum L.)?. Naturwissenschaften.

[CR58] Park S, Kang K (2009). Induction of serotonin biosynthesis is uncoupled from the coordinated induction of tryptophan biosynthesis in pepper fruits (Capsicum annuum) upon pathogen infection. Planta.

[CR59] Park S, Kang K (2011). Production of serotonin by dual expression of tryptophan decarboxylase and tryptamine 5-hydroxylase in Escherichia coli. Appl Microbiol Biotechnol.

[CR60] Park S, Lee K (2012). Tryptamine 5-hydroxylase-deficient Sekiguchi rice induces synthesis of 5-hydroxytryptophan and N-acetyltryptamine but decreases melatonin biosynthesis during senescence process of detached leaves. J Pineal Res.

[CR61] Penfield S, Hall A (2009). A role for multiple circadian clock genes in the response to signals that break seed dormancy in Arabidopsis. Plant Cell.

[CR62] Poulsen C, Bongaerts RJ (1993). Purification and characterization of anthranilate synthase from Catharanthus roseus. Eur J Biochem.

[CR63] Radwanski ER, Last RL (1995). Tryptophan biosynthesis and metabolism: biochemical and molecular genetics. Plant Cell.

[CR64] Romero RM, Roberts MF (1995). Anthranilate synthase in microorganisms and plants. Phytochemistry.

[CR65] Roshchina VV (2001). Neurotransmitters in Plant Life.

[CR66] Saha R, Suthers PF (2011). Zea mays iRS1563: a comprehensive genome-scale metabolic reconstruction of maize metabolism. PLoS One.

[CR67] Schutz A, Golbik R (2003). Studies on structure-function relationships of indolepyruvate decarboxylase from Enterobacter cloacae, a key enzyme of the indole acetic acid pathway. Eur J Biochem.

[CR68] Seo YS, Chern M (2011). Towards establishment of a rice stress response interactome. PLoS Genet.

[CR69] Shimura K, Okada A (2007). Identification of a biosynthetic gene cluster in rice for momilactones. J Biol Chem.

[CR70] Small I, Peeters N (2004). Predotar: A tool for rapidly screening proteomes for N-terminal targeting sequences. Proteomics.

[CR71] Song WY, Wang GL (1995). A receptor kinase-like protein encoded by the rice disease resistance gene, Xa21. Science.

[CR72] Sonnhammer EL, von Heijne G (1998). A hidden Markov model for predicting transmembrane helices in protein sequences. Proc Int Conf Intell Syst Mol Biol.

[CR73] Stepanova AN, Robertson-Hoyt J (2008). TAA1-mediated auxin biosynthesis is essential for hormone crosstalk and plant development. Cell.

[CR74] Stromback L, Lambrix P (2005). Representations of molecular pathways: an evaluation of SBML, PSI MI and BioPAX. Bioinformatics.

[CR75] Tao Y, Ferrer JL (2008). Rapid synthesis of auxin via a new tryptophan-dependent pathway is required for shade avoidance in plants. Cell.

[CR76] Tokunaga H, Kojima M (2012). Arabidopsis lonely guy (LOG) multiple mutants reveal a central role of the LOG-dependent pathway in cytokinin activation. Plant J.

[CR77] Urbanczyk-Wochniak E, Sumner LW (2007). MedicCyc: a biochemical pathway database for Medicago truncatula. Bioinformatics.

[CR78] Wakasa K, Hasegawa H (2006). High-level tryptophan accumulation in seeds of transgenic rice and its limited effects on agronomic traits and seed metabolite profile. J Exp Bot.

[CR79] Wang GL, Song WY (1996). The cloned gene, Xa21, confers resistance to multiple Xanthomonas oryzae pv. oryzae isolates in transgenic plants. Mol Plant Microbe Interact.

[CR80] Wang W, Barnaby JY (2011). Timing of plant immune responses by a central circadian regulator. Nature.

[CR81] Wilderman PR, Xu M (2004). Identification of syn-pimara-7,15-diene synthase reveals functional clustering of terpene synthases involved in rice phytoalexin/allelochemical biosynthesis. Plant Physiol.

[CR82] Won C, Shen X (2011). Conversion of tryptophan to indole-3-acetic acid by TRYPTOPHAN AMINOTRANSFERASES OF ARABIDOPSIS and YUCCAs in Arabidopsis. Proc Natl Acad Sci USA.

[CR83] Xie G, Forst C (2002). "Significance of two distinct types of tryptophan synthase beta chain in Bacteria, Archaea and higher plants". Genome Biol.

[CR84] Xu M, Hillwig ML (2004). Functional identification of rice syn-copalyl diphosphate synthase and its role in initiating biosynthesis of diterpenoid phytoalexin/allelopathic natural products. Plant J.

[CR85] Xu M, Wilderman PR (2007). Functional characterization of the rice kaurene synthase-like gene family. Phytochemistry.

[CR86] Xu W, Yang R (2011). Transcriptome phase distribution analysis reveals diurnal regulated biological processes and key pathways in rice flag leaves and seedling leaves. PLoS One.

[CR87] Yamazaki Y, Jaiswal P (2005). Biological ontologies in rice databases. An introduction to the activities in Gramene and Oryzabase. Plant Cell Physiol.

[CR88] Yoshimura S, Yamanouchi U (1998). Expression of Xa1, a bacterial blight-resistance gene in rice, is induced by bacterial inoculation. Proc Natl Acad Sci USA.

[CR89] Youens-Clark K, Buckler E (2011). "Gramene database in 2010: updates and extensions". Nucleic Acids Res.

[CR90] Yu HT, Xu SB (2011). Comparative Proteomic Study Reveals the Involvement of Diurnal Cycle in Cell Division, Enlargement, and Starch Accumulation in Developing Endosperm of Oryza sativa. J Proteome Res.

[CR91] Yuan Q, Ouyang S (2005). The institute for genomic research Osa1 rice genome annotation database. Plant Physiol.

[CR92] Yun KY, Park MR (2010). Transcriptional regulatory network triggered by oxidative signals configures the early response mechanisms of japonica rice to chilling stress. BMC Plant Biol.

[CR93] Zhang P, Dreher K (2010). Creation of a genome-wide metabolic pathway database for Populus trichocarpa using a new approach for reconstruction and curation of metabolic pathways for plants. Plant Physiol.

[CR94] Zhang P, Foerster H (2005). MetaCyc and AraCyc. Metabolic pathway databases for plant research. Plant Physiol.

[CR95] Zhang PG, Huang SZ (2010). Extensive divergence in alternative splicing patterns after gene and genome duplication during the evolutionary history of Arabidopsis. Mol Biol Evol.

[CR96] Zhao J, Last RL (1996). Coordinate regulation of the tryptophan biosynthetic pathway and indolic phytoalexin accumulation in Arabidopsis. Plant Cell.

[CR97] Zhao Y (2010). Auxin biosynthesis and its role in plant development. Annu Rev Plant Biol.

[CR98] Zhao Y (2012). Auxin Biosynthesis: A Simple Two-Step Pathway Converts Tryptophan to Indole-3-Acetic Acid in Plants. Mol Plant.

[CR99] Zimmermann P, Hennig L (2005). Gene-expression analysis and network discovery using Genevestigator. Trends Plant Sci.

[CR100] Zimmermann P, Hirsch-Hoffmann M (2004). GENEVESTIGATOR. Arabidopsis microarray database and analysis toolbox. Plant Physiol.

