# Supplementary figures and images for: A genome scale metabolic network for rice and accompanying analysis of tryptophan, auxin and serotonin biosynthesis regulation under biotic stress
Source: Rice (N Y). 2013 May 29;6:15. doi: 10.1186/1939-8433-6-15 (PMC4883713; doi:10.1186/1939-8433-6-15)

## Slide 1
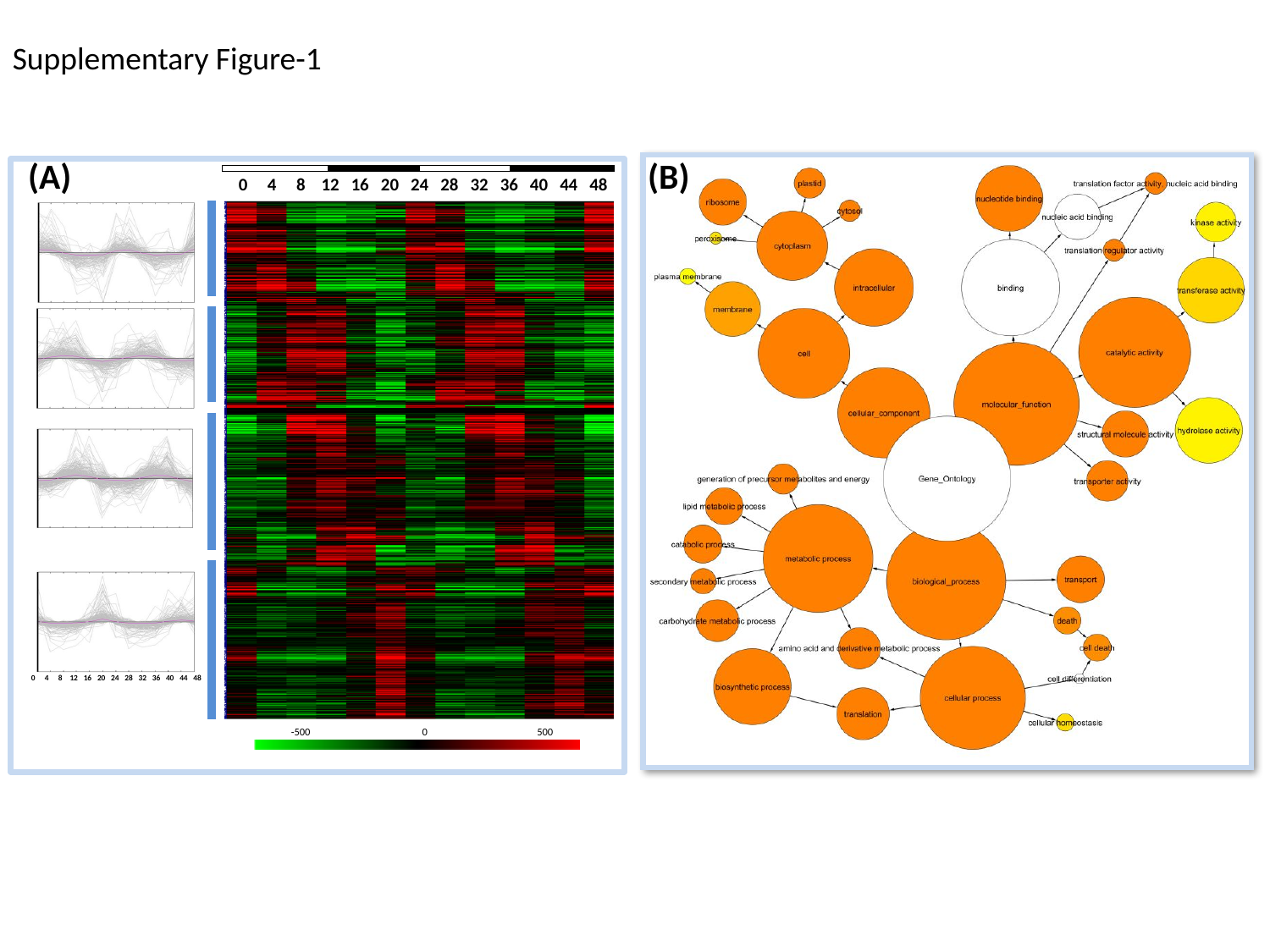

Supplementary Figure-1
(A)
(B)
 0 4 8 12 16 20 24 28 32 36 40 44 48
0 4 8 12 16 20 24 28 32 36 40 44 48
-500 0 500

Supplement: Supplementary file 3 — Additional file 3: Figure S1: Expression pattern and over-represented metabolic processes of diurnally cycling genes. (A) Hierarchical clustering of gene expression pattern of diurnally cycling genes in rice, mapping to RiceCyc metabolic pathways. Mean centered expression levels are represented with green denoting downregulation, and red indicating upregulation. (B) Over-represented gene ontology categories of gene set represented in (A). (PPTX 505 KB) [file 12284_2013_52_MOESM3_ESM.pptx]

## Slide 1
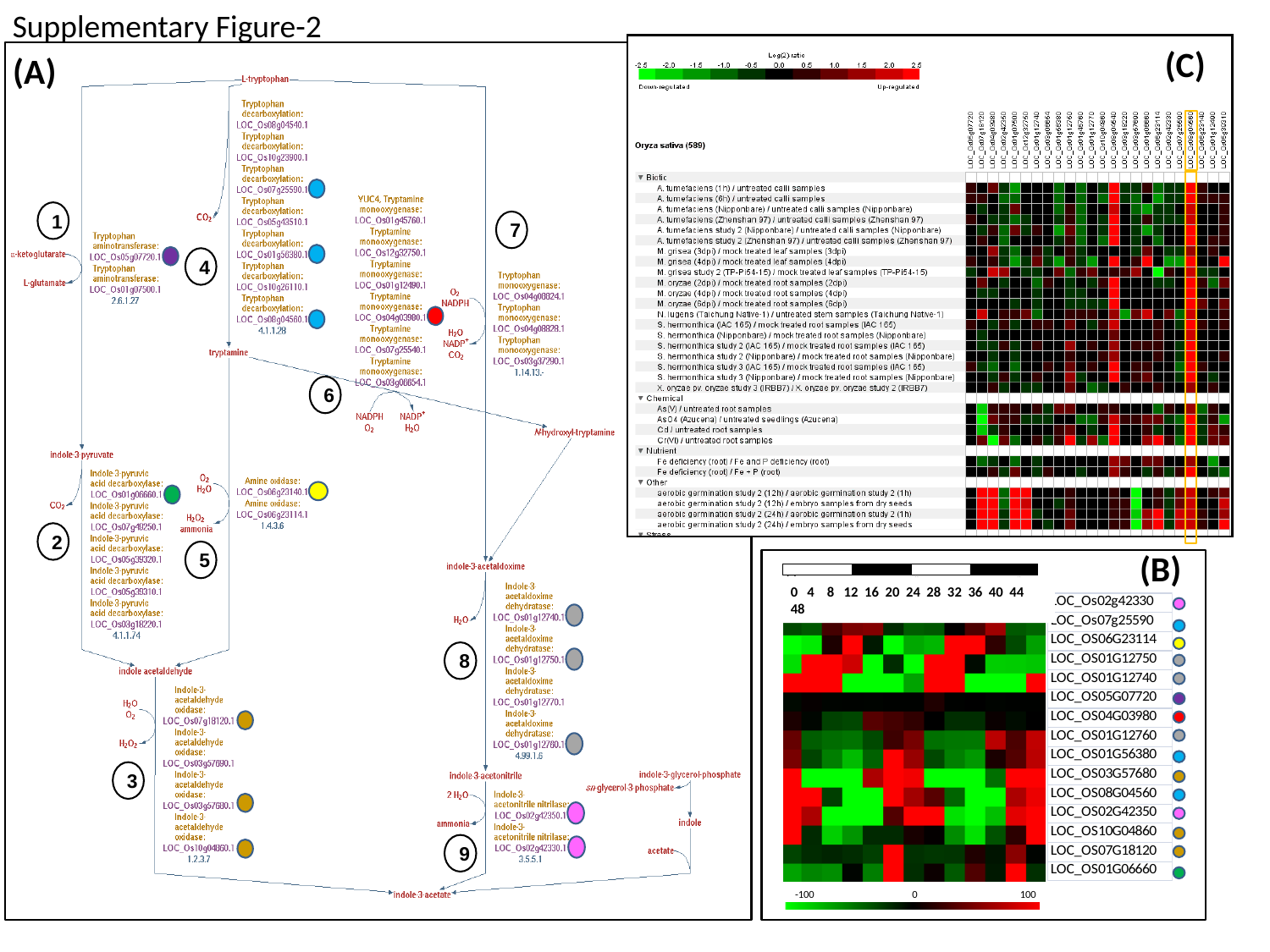

Supplementary Figure-2
(C)
(A)
1
7
4
6
2
5
8
3
9
(B)
-100 0 100
0 4 8 12 16 20 24 28 32 36 40 44 48

Supplement: Supplementary file 4 — Additional file 4: Figure S2: Gene expression analysis of the genes associated with enzymes catalyzing IAA biosynthesis. (A) A zoom-in view of IAA biosynthetic pathway. (B) Heat map of expression patterns of all cycling genes in the pathway with the gene ID and reaction of the pathway. Colored circles are used to identify individual reactions. (C) Biotic and abiotic stress induction of tryptophan biosynthetic genes based on data compiled in Genevestigator for rice. Expression data available for perturbations were filtered for significance level (p- value <0.05) and fold change (>2, based on gene in column with a yellow outline). (PPTX 221 KB) [file 12284_2013_52_MOESM4_ESM.pptx]

## Slide 1
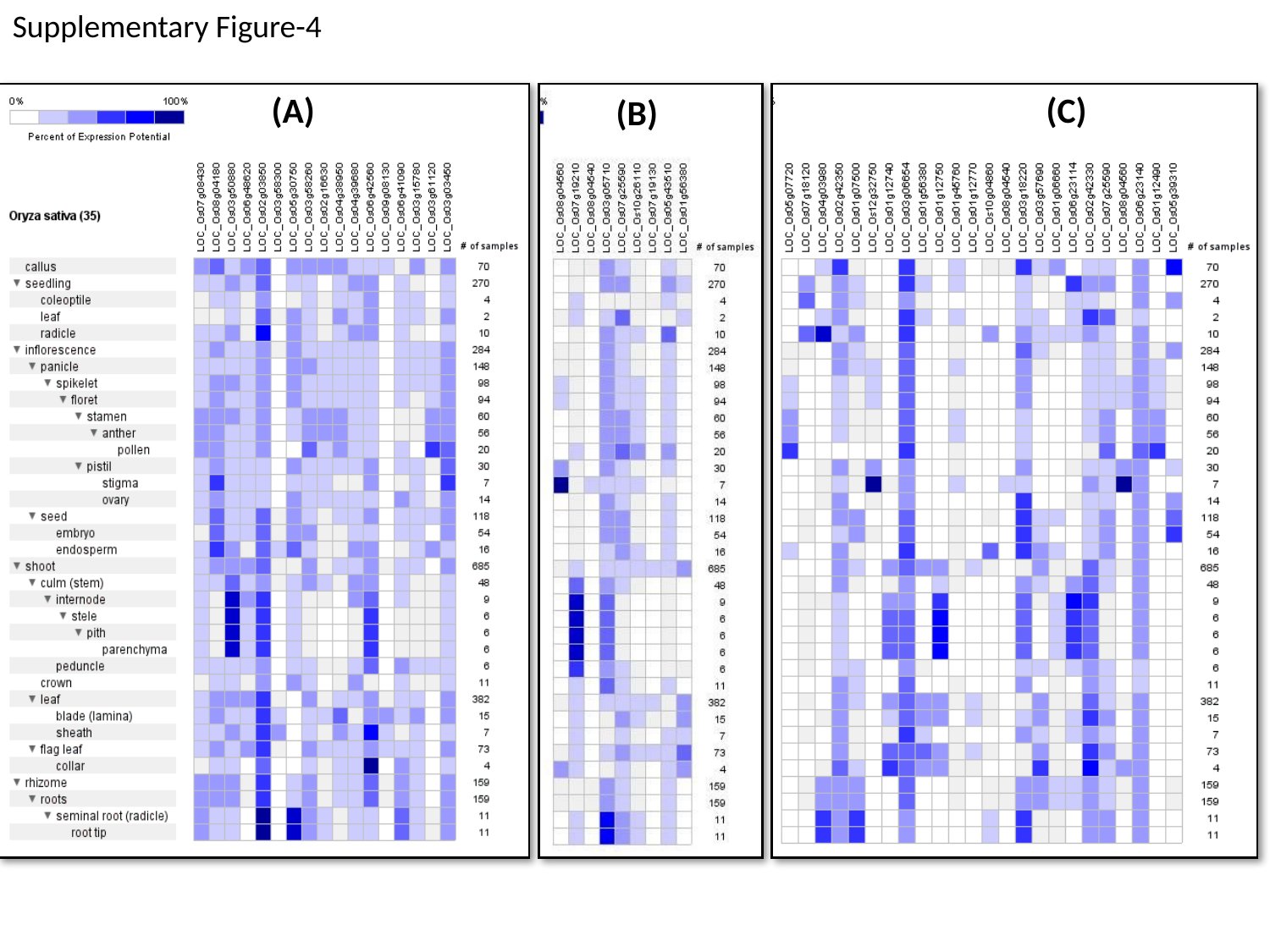

Supplementary Figure-4
(A)
(C)
(B)

Supplement: Supplementary file 5 — Additional file 5: Figure S4: Tissue type expression pattern of genes in tryptophan biosynthesis (A), serotonin biosynthesis (B), and IAA biosynthesis (C) from data compiled in Genevestigator. (PPTX 178 KB) [file 12284_2013_52_MOESM5_ESM.pptx]

**(A)**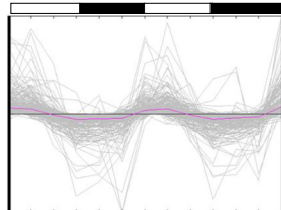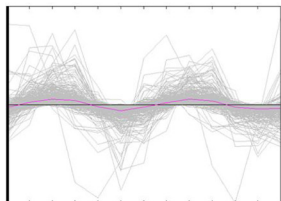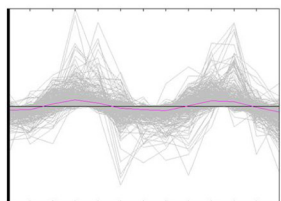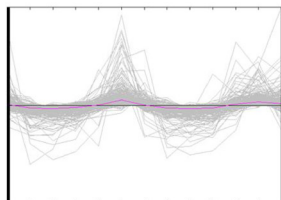

0 4 8 12 16 20 24 28 32 36 40 44 48

**(B)**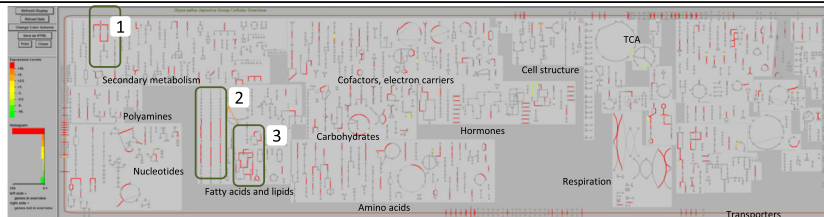

0h

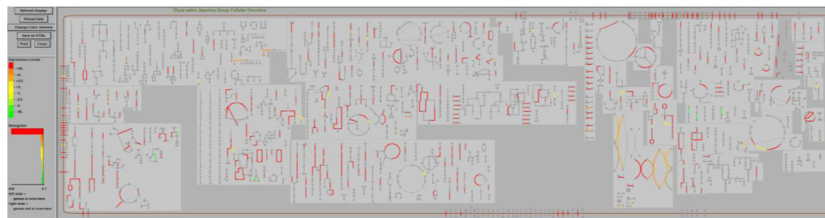

8h

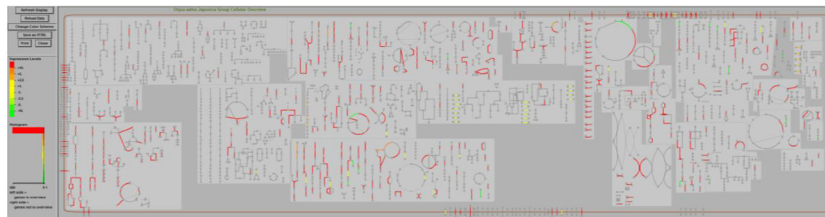

12h

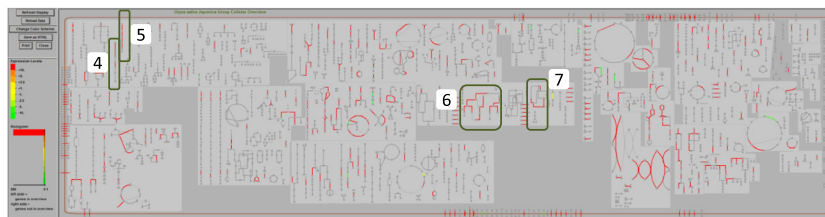

20h

Supplement: Supplementary file 10 — Authors’ original file for figure 3 [file 12284_2013_52_MOESM10_ESM.pdf]

(A)

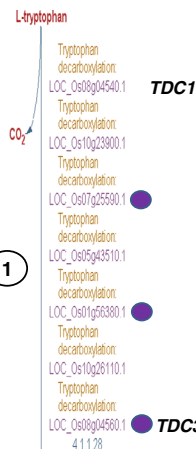

(B)

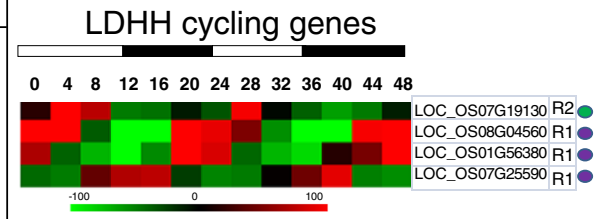

(C)

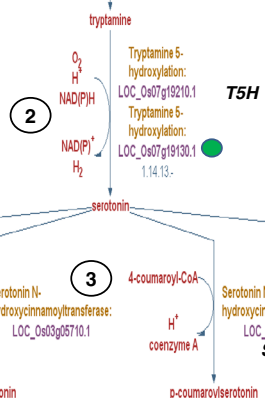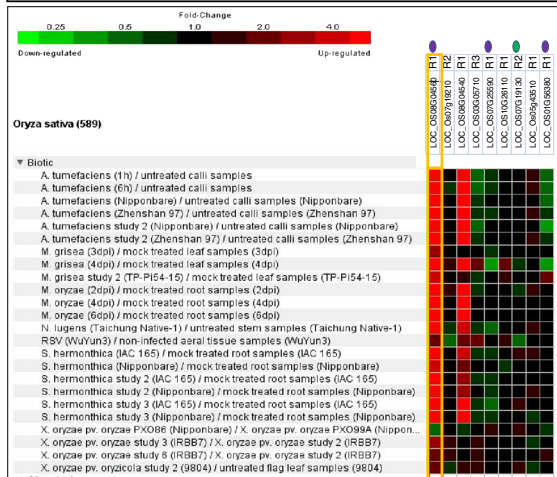

Supplement: Supplementary file 11 — Authors’ original file for figure 4 [file 12284_2013_52_MOESM11_ESM.pdf]

## Slide 1
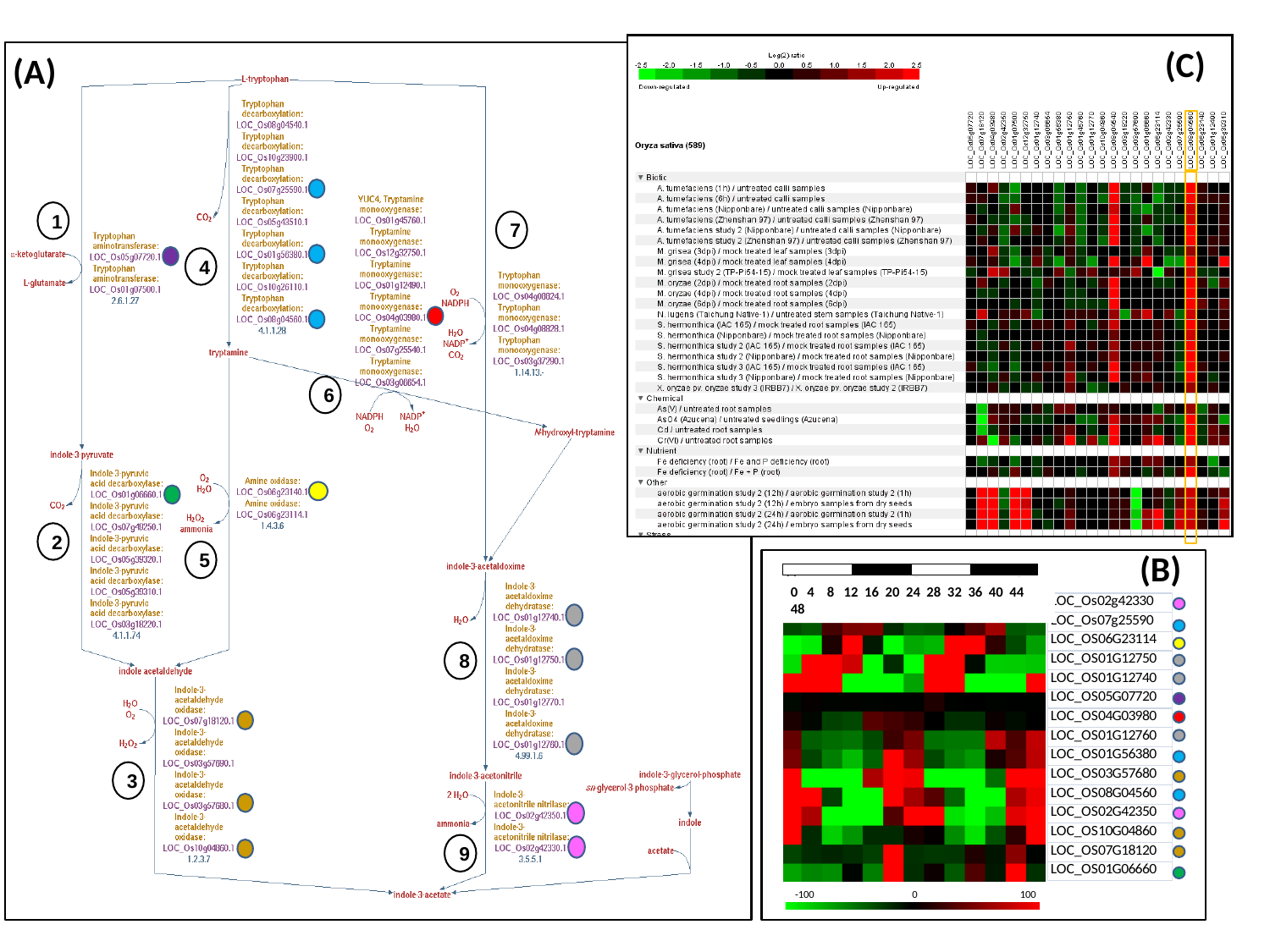

(C)
(A)
1
7
4
6
2
5
8
3
9
(B)
-100 0 100
0 4 8 12 16 20 24 28 32 36 40 44 48

Supplement: Supplementary file 12 — Authors’ original file for figure 5 [file 12284_2013_52_MOESM12_ESM.pptx]
