# Supplementary material for: A genome scale metabolic network for rice and accompanying analysis of tryptophan, auxin and serotonin biosynthesis regulation under biotic stress
Source: Rice (N Y). 2013 May 29;6:15. doi: 10.1186/1939-8433-6-15 (PMC4883713; doi:10.1186/1939-8433-6-15)
Supplement: Supplementary file 6 — Additional file 6: Figure S3: Rice tryptophan biosynthetic enzyme interactome based on RiceNet. Blue circles on the periphery of the interactome represent tryptophan biosynthetic enzymes. Green circles represent interacting proteins identified from RiceNet. Three of the five anthranilate phosphoribosyltransferase paralogs (LOC_Os06g41090, LOC_Os04g39680, LOC_Os05g30750) did not share interactions with other tryptophan biosynthetic enzymes and clustered separately (lower left corner of Figure). (PPTX 2 MB) [file 12284_2013_52_MOESM6_ESM.pptx]

## Slide 1
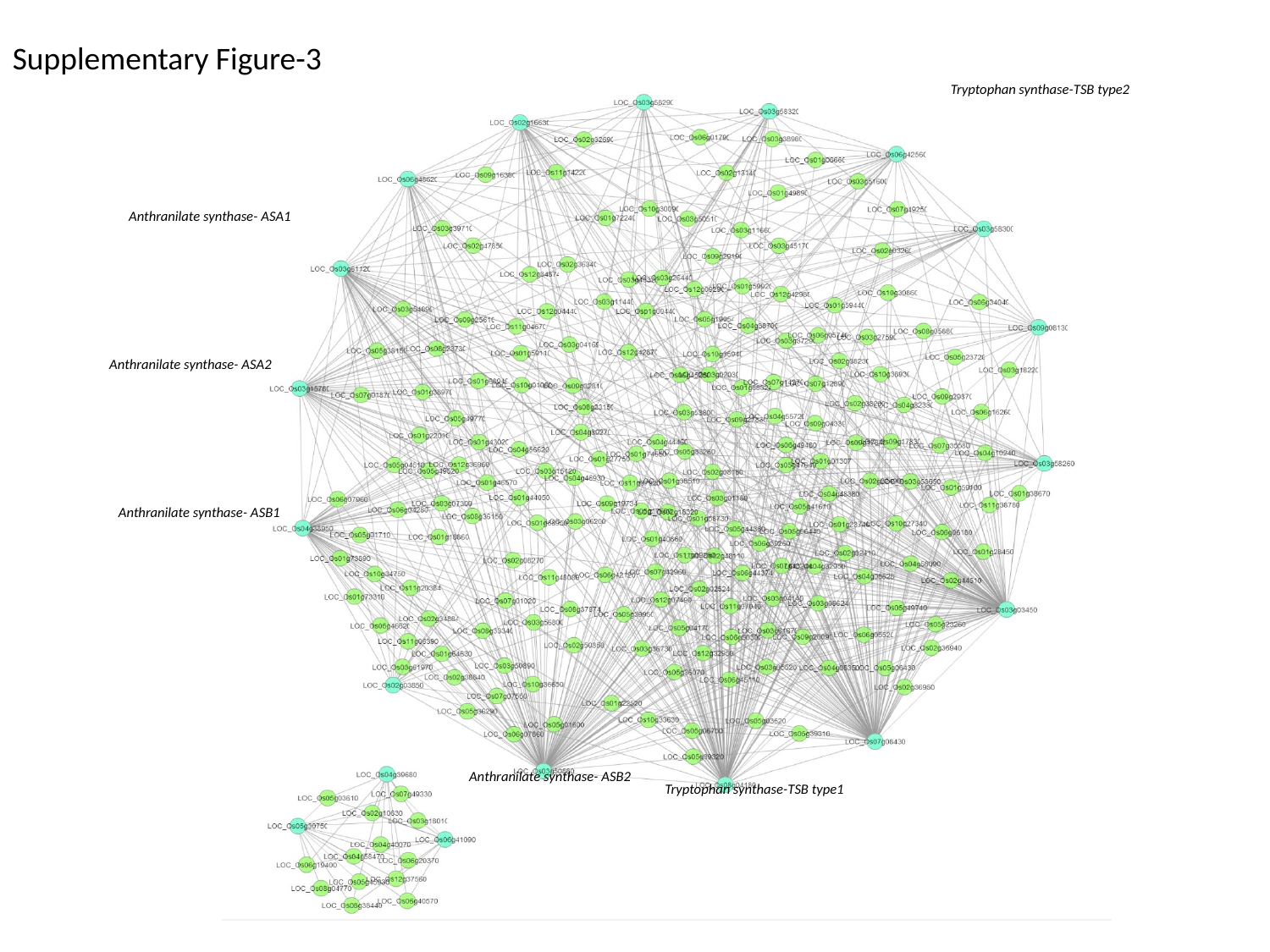

Supplementary Figure-3
Tryptophan synthase-TSB type2
Anthranilate synthase- ASA1
Anthranilate synthase- ASA2
Anthranilate synthase- ASB1
Anthranilate synthase- ASB2
Tryptophan synthase-TSB type1
