# Supplementary material for: A genome scale metabolic network for rice and accompanying analysis of tryptophan, auxin and serotonin biosynthesis regulation under biotic stress
Source: Rice (N Y). 2013 May 29;6:15. doi: 10.1186/1939-8433-6-15 (PMC4883713; doi:10.1186/1939-8433-6-15)
Supplement: Supplementary file 8 — Authors’ original file for figure 1 [file 12284_2013_52_MOESM8_ESM.pdf]

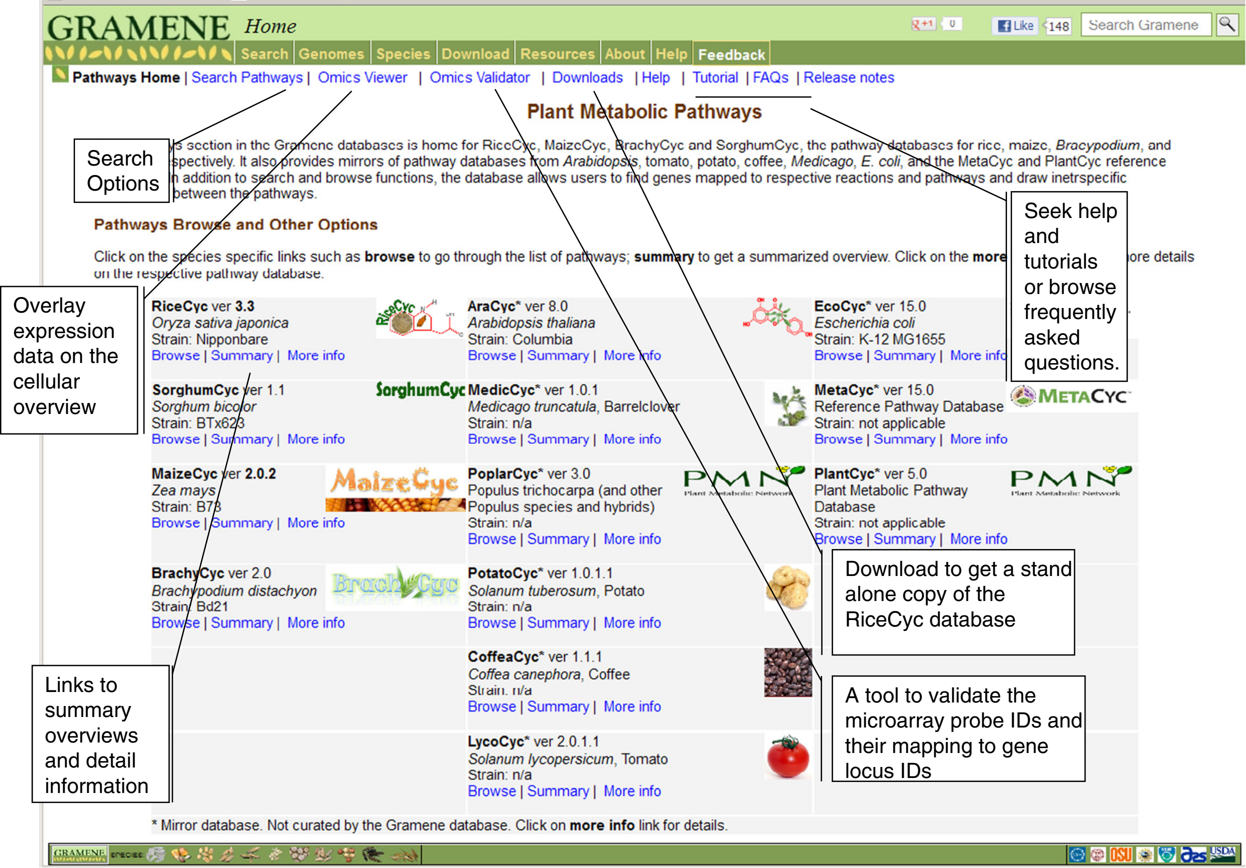
GRAMENE Home
Search Gramene
Search | Genomes | Species | Download | Resources | About | Help | Feedback
Pathways Home | Search Pathways | Omics Viewer | Omics Validator | Downloads | Help | Tutorial | FAQs | Release notes
Plant Metabolic Pathways
s section in the Gramene databases is home for RiceCyc, MaizeCyc, BrachyCyc and SorghumCyc, the pathway databases for rice, maize, Bracypodium, and
spectively. It also provides mirrors of pathway databases from Arabidopsis, tomato, potato, coffee, Medicago, E. coli, and the MetaCyc and PlantCyc reference
n addition to search and browse functions, the database allows users to find genes mapped to respective reactions and pathways and draw inetrspecific
between the pathways.
Pathways Browse and Other Options
Click on the species specific links such as browse to go through the list of pathways; summary to get a summarized overview. Click on the more
ore details
on the respective pathway database.
RiceCyc ver 3.3
Oryza sativa japonica
Strain: Nipponbare
Browse | Summary | More info
AraCyc* ver 8.0
Arabidopsis thaliana
Strain: Columbia
Browse | Summary | More info
EcoCyc* ver 15.0
Escherichia coli
Strain: K-12 MG1655
Browse | Summary | More info
SorghumCyc ver 1.1
Sorghum bicolor
Strain: BTx623
Browse | Summary | More info
MedicCyc* ver 1.0.1
Medicago truncatula, Barrelclover
Strain: n/a
Browse | Summary | More info
MetaCyc* ver 15.0
Reference Pathway Database
Strain: not applicable
Browse | Summary | More info
MaizeCyc ver 2.0.2
Zea mays
Strain: B73
Browse | Summary | More info
PoplarCyc* ver 3.0
Populus trichocarpa (and other Populus species and hybrids)
Strain: n/a
Browse | Summary | More info
PlantCyc* ver 5.0
Plant Metabolic Pathway Database
Strain: not applicable
Browse | Summary | More info
BrachyCyc ver 2.0
Brachypodium distachyon
Strain: Bd21
Browse | Summary | More info
PotatoCyc* ver 1.0.1.1
Solanum tuberosum, Potato
Strain: n/a
Browse | Summary | More info
CoffeaCyc* ver 1.1.1
Coffea canephora, Coffee
Strain: n/a
Browse | Summary | More info
LycoCyc* ver 2.0.1.1
Solanum lycopersicum, Tomato
Strain: n/a
Browse | Summary | More info
* Mirror database. Not curated by the Gramene database. Click on more info link for details.
Search Options
Overlay expression data on the cellular overview
Links to summary overviews and detail information
Seek help and tutorials or browse frequently asked questions.
Download to get a stand alone copy of the RiceCyc database
A tool to validate the microarray probe IDs and their mapping to gene locus IDs
