# Supplementary material for: A genome scale metabolic network for rice and accompanying analysis of tryptophan, auxin and serotonin biosynthesis regulation under biotic stress
Source: Rice (N Y). 2013 May 29;6:15. doi: 10.1186/1939-8433-6-15 (PMC4883713; doi:10.1186/1939-8433-6-15)
Supplement: Supplementary file 9 — Authors’ original file for figure 2 [file 12284_2013_52_MOESM9_ESM.pdf]

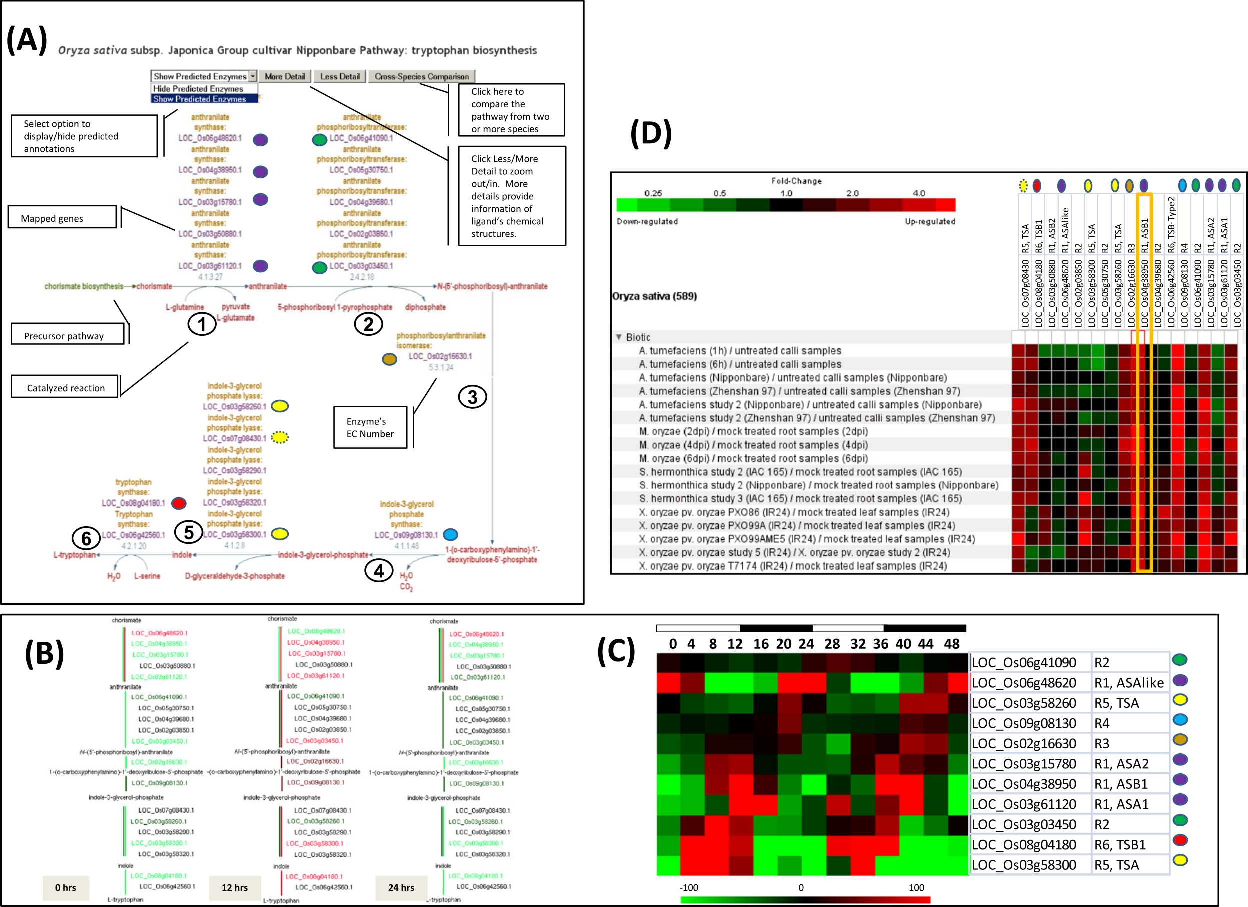
(A)
Oryza sativa subsp. Japonica Group cultivar Nipponbare Pathway: tryptophan biosynthesis
Show Predicted Enzymes
More Detail
Less Detail
Cross-Species Comparison
Hide Predicted Enzymes
Show Predicted Enzymes
Click here to compare the pathway from two or more species
Click Less/More Detail to zoom out/in. More details provide information of ligand's chemical structures.
Select option to display/hide predicted annotations
Mapped genes
Precursor pathway
Catalyzed reaction
Enzyme's EC Number
anthranilate synthase: LOC_Os06g48620.1
anthranilate synthase: LOC_Os04g38950.1
anthranilate synthase: LOC_Os03g15780.1
anthranilate synthase: LOC_Os03g50880.1
anthranilate synthase: LOC_Os03g61120.1
4.1.3.27
anthranilate phosphoribosyltransferase: LOC_Os06g41090.1
anthranilate phosphoribosyltransferase: LOC_Os05g30750.1
anthranilate phosphoribosyltransferase: LOC_Os04g39680.1
anthranilate phosphoribosyltransferase: LOC_Os02g03850.1
anthranilate phosphoribosyltransferase: LOC_Os03g03450.1
2.4.2.18
chorismate biosynthesis
chorismate
anthranilate
N-(5'-phosphoribosyl)-anthranilate
L-glutamine
pyruvate
L-glutamate
5-phosphoribosyl 1-pyrophosphate
diphosphate
1
2
3
4
5
6
phosphoribosylanthranilate isomerase: LOC_Os02g16630.1
5.3.1.24
indole-3-glycerol phosphate lyase: LOC_Os03g58260.1
indole-3-glycerol phosphate lyase: LOC_Os07g08430.1
indole-3-glycerol phosphate lyase: LOC_Os03g58290.1
indole-3-glycerol phosphate lyase: LOC_Os03g58320.1
indole-3-glycerol phosphate lyase: LOC_Os03g58300.1
4.1.2.8
tryptophan synthase: LOC_Os08g04180.1
Tryptophan synthase: LOC_Os06g42560.1
4.2.1.20
indole-3-glycerol phosphate synthase: LOC_Os09g08130.1
4.1.1.48
L-tryptophan
indole
indole-3-glycerol-phosphate
1-(o-carboxyphenylamino)-1'-deoxyribulose-5'-phosphate
H2O
L-serine
D-glyceraldehyde-3-phosphate
H2O
CO2
(B)
0 hrs
12 hrs
24 hrs
chorismate
anthranilate
N-(5'-phosphoribosyl)-anthranilate
1-(o-carboxyphenylamino)-1'-deoxyribulose-5'-phosphate
indole-3-glycerol-phosphate
indole
L-tryptophan
(C)
0 4 8 12 16 20 24 28 32 36 40 44 48
LOC_Os06g41090 R2
LOC_Os06g48620 R1, ASAlike
LOC_Os03g58260 R5, TSA
LOC_Os09g08130 R4
LOC_Os02g16630 R3
LOC_Os03g15780 R1, ASA2
LOC_Os04g38950 R1, ASB1
LOC_Os03g61120 R1, ASA1
LOC_Os03g03450 R2
LOC_Os08g04180 R6, TSB1
LOC_Os03g58300 R5, TSA
-100
0
100
(D)
Fold-Change
0.25
0.5
1.0
2.0
4.0
Down-regulated
Up-regulated
Oryza sativa (589)
LOC_Os07g08430 R5, TSA
LOC_Os08g04180 R6, TSB1
LOC_Os03g50880 R1, ASB2
LOC_Os06g48620 R1, ASAlike
LOC_Os02g03850 R2
LOC_Os03g58300 R5, TSA
LOC_Os05g30750 R2
LOC_Os03g58260 R5, TSA
LOC_Os02g16630 R3
LOC_Os04g38950 R1, ASB1
LOC_Os04g39680 R2
LOC_Os06g42560 R6, TSB-Type2
LOC_Os09g08130 R4
LOC_Os06g41090 R2
LOC_Os03g15780 R1, ASA2
LOC_Os03g61120 R1, ASA1
LOC_Os03g03450 R2
Biotic
A. tumefaciens (1h) / untreated calli samples
A. tumefaciens (6h) / untreated calli samples
A. tumefaciens (Nipponbare) / untreated calli samples (Nipponbare)
A. tumefaciens (Zhenshan 97) / untreated calli samples (Zhenshan 97)
A. tumefaciens study 2 (Nipponbare) / untreated calli samples (Nipponbare)
A. tumefaciens study 2 (Zhenshan 97) / untreated calli samples (Zhenshan 97)
M. oryzae (2dpi) / mock treated root samples (2dpi)
M. oryzae (4dpi) / mock treated root samples (4dpi)
M. oryzae (6dpi) / mock treated root samples (6dpi)
S. hermonthica study 2 (IAC 165) / mock treated root samples (IAC 165)
S. hermonthica study 2 (Nipponbare) / mock treated root samples (Nipponbare)
S. hermonthica study 3 (IAC 165) / mock treated root samples (IAC 165)
X. oryzae pv. oryzae PXO86 (IR24) / mock treated leaf samples (IR24)
X. oryzae pv. oryzae PXO99A (IR24) / mock treated leaf samples (IR24)
X. oryzae pv. oryzae PXO99AME5 (IR24) / mock treated leaf samples (IR24)
X. oryzae pv. oryzae study 5 (IR24) / X. oryzae pv. oryzae study 2 (IR24)
X. oryzae pv. oryzae T7174 (IR24) / mock treated leaf samples (IR24)
