# Supplementary material for: A genome scale metabolic network for rice and accompanying analysis of tryptophan, auxin and serotonin biosynthesis regulation under biotic stress
Source: Rice (N Y). 2013 May 29;6:15. doi: 10.1186/1939-8433-6-15 (PMC4883713; doi:10.1186/1939-8433-6-15)
Supplement: Supplementary file 13 — Authors’ original file for figure 6 [file 12284_2013_52_MOESM13_ESM.pdf]

Response to Reviewers

Manuscript # 1624295146853521

General note: The major changes are highlighted in red colored text in the manuscript.

Reviewer's report
Title: Genome Scale metabolic network for rice and analysis of tryptophan and derivative biosynthesis regulation during biotic stress
Version: 3 **Date:** 18 December 2012
Reviewer number: 1
Reviewer's report:
The paper reports RiceCyc, an integration of a metabolic pathway tools with rice gene functional and structural annotation. It uses a well-established tool, PathoLogic Pathway Predictor, adding to many species with sequenced genomes that have used this strategy to represent metabolism. This tool has specific value to rice researchers as it couples some very nice analysis tools that can be used to study gene expression results and other data, as well as depict on the genome glyphs, locations of specific pathways on the genome.
==========================
MAJOR ISSUES to address.
RE Content of RiceCyc database.
(1) Provide some understanding of computational accuracy of the largely predicted representation. For example, overall statistics on numbers of gene models assigned to metabolic reactions, and those that are not, and the expectations for an organism might be helpful. Presumably there are reviews on rice and Arabidopsis genome annotation the authors could refer to.

We have included a paragraph in the section ‘Development of RiceCyc pathway database’ to address the question of computational accuracy. We just received notice that our work on MaizeCyc, a maize metabolic network built using the same workflow as RiceCyc, has been accepted for publication by the journal Plant Genome. Similar to maize specifically, and other reports we expect rice metabolic genes to comprise ~15-20% of the total gene set. This percentage may increase based on ancestral genome duplications and the ploidy of a given species. For example, the grape and eucalyptus metabolic networks (under preparation in our lab) show a higher percentage (~30%) of the total gene loci mapping to metabolic annotations.

(2) Provide a list of pathways that have been curated, and numbers of reactions curated. This might be in a supplemental file if very large.

We appreciate the reviewer’s suggestion. We have provided a URL in the manuscript that directs the reader to more information on curated pathways in each release. The pathway database page (http://gramene.org/pathway/ricecyc.html) contains a

“Modifications” hyperlink, which leads to a page (http://gramene.org/pathway/ricecyc_deleted_pathways.html) enumerating a list of pathways and reactions that have undergone some kind of editing.

(3) Provide a list of pathways added to RiceCyc that are unique to rice, and represented with experimental evidence at RiceCyc.

We appreciate the reviewer’s suggestion. We provide text of this nature in the description comments of the pathway/reaction/small molecule when applicable, however, it can be difficult to comprehensively evaluate the uniqueness of all rice pathways; an attempt to do so may mislead users. Identifying pathway uniqueness in a species depends on several factors: (1) revisions and annotations as a continuing process, (2) the amount of curation not only in rice but in the comparative species datasets which we do not curate/maintain and (3) a deficiency of experimental evidence and lack of published reports. Regardless, we have changed the language in the abstract from “rice specific” to “pathways reported in rice”. We prefer to enable users to explore inter-species pathway differences on their own. The RiceCyc web tool allows this comparison between any two or more species (http://pathway.gramene.org/comp-genomics), as mentioned in the manuscript. This tool provides several options and data types to choose from.

(4) Describe the accuracy of the RiceCyc pipeline relative to data drawn from other resources, such as the KEGG and MetaCyc.

We appreciate the reviewer’s suggestion. Based on our workflow, we have found that it is fairly consistent, because much of it depends on the homology and presence of domains. Since our homology-based predictions are not simply based on the pairwise matching but a kingdom-wide phylogenetic placement of genes in gene families, it has a bit more confidence. There is a pitfall to this method in that sometimes one can get an overly excessive number of family members. However, we have tried to filter those orthologs from the gene trees that do not match >=50% ID matches in reciprocal BLASTP results. KEGG and MetaCyc annotations were accurate.

Are there any plans to keep the dataset updated in the future?

We appreciate the reviewer’s concerns and yes, we do plan to keep the databases updated in the future. We were recently funded for the next 5 years, allowing us to keep these resources up to date. However, we would like to note that since Ptools-based networks such as RiceCyc are not best-suited to handle the integration of regulatory pathways with metabolic and transport pathways, we are developing a Plant Reactome to cater to this regulatory pathways deficiency. The metabolic network from RiceCyc will be represented in the Plant Reactome to provide a more complete picture. Regardless, RiceCyc *will* be maintained as a user resource.

Curiously, one of the pathways used as

example, momilactone biosynthesis, is missing data currently available at both MetaCyc and the KEGG. For example, that a momilactone A biosynthesis enzyme uses NAD[P] as a reactant, and has been assigned an EC number, 1.1.1.295.

Thank you for bringing this to our attention. We have updated this annotation and it will be made available to the public in the next database release. At the time of curation, this particular pathway was not assigned an EC number; as you have pointed out, it is now available. On the other hand, the gene was correctly associated to the reaction and the pathway, which we view is a biologically significant piece of information. Annotations can be improved as we learn of new reports; it is an ongoing process. We encourage user community participation in reporting feedback on deficiencies and inaccuracies so that we can help build better annotations in the RiceCyc database.

(5) Does RiceCyc/Gramene curation lead to adding any information to more central resources such as GO, MetaCyc or KEGG. MetaCyc recently reports updating secondary plant metabolic pathways, but does not include the rice specific pathways referred to in this report.
RE Use Cases

Since much of this has to be changed at the reference library level in KEGG and MetaCyc, we prefer delegating the responsibility to these resources. However, we do report and collaborate with the GO and MetaCyc annotation groups. At times, when we find there is a need, we work with them by either asking for new GO terms and/or revising the definitions and cross-references. We share our new/revised pathway and reaction annotations with the MetaCyc group to facilitate addition to their reference data set. We do not anticipate a dedicated effort towards that goal as we are limited by our resources and are not funded to do so.

(6) It is not clear that the extensive use cases, and supplemental data files, largely available from other sources, add to the paper. Pathway Tool suite papers can be referred to for use cases. The provided brief summaries of tool functions could be made more clear as to whether one needs to download a copy of the database for best function.

We appreciate the reviewer’s comments. It is true that the supplementary data files are derived from data available in other sources but a user would have to spend time aggregating and processing this data in order to reference it while reading our paper. Unless the editor feels otherwise we feel that it is beneficial for the reader to have immediate access to the specific data that is usedd in this manuscript.

We also think that this is an opportunity for us to showcase our research activity and the power of using such tools for building hypothesis-driven research. There are lots of tools in the bioinformatics arena, of which many are very well-developed for browsing and searching, but they typically have fewer analytical functionalities. RiceCyc allows users

to analyze data in the context of the plant's biology, in addition to searching/browsing the data. Our study shows what a user can accomplish using the tool and publicly available data sets.

We do not see the biology described here as simple “use cases”, hence we would prefer to keep it in the manuscript in order to highlight RiceCyc's utility for plant biology researchers. If suggested otherwise, we will be willing to remove it. Needless to say, the biology described in this manuscript is the first report where bioinformatic analysis suggests that there is coordinated regulation of tryptophan, auxin and serotonin metabolism under response to pathogen challenge and diurnal photoperiod treatments. Our feeling is that this is an important milestone and the manuscript, if accepted for publication, would be beneficial to rice researchers and the Journal. However, we do agree that it is still a proof-of-concept model.

========================
Minor essential elements.
-- The 2nd sentence of the introduction is misleading. It seems unaware that rice functional annotation, as is the case for most angiosperms, largely relies on extensive Arabiodopsis research and literature curation. The functional data for rice is extremely valuable, especially for related species, but unfortunately there are few resources for literature curation these days.

We have modified the text as suggested.

-- The legends for the supplemental tables 1, 2 are inadequate. They do not to describe the content of each field.

We apologize for this oversight. We have elaborated the supplementary table legends to better describe the contents.

Level of interest: An article whose findings are important to those with closely related research interests

Quality of written English: Needs some language corrections before being Published

We have updated the manuscript accordingly as suggested by the reviewer. Two people did the copy editing.

Reviewer's report
Title: Genome Scale metabolic network for rice and analysis of tryptophan and derivative biosynthesis regulation during biotic stress
Version: 3 **Date:** 31 December 2012
Reviewer number: 2
Reviewer's report:
This manuscript report a development of RiceCyc database and its application to gene expression analysis related to immune response and diurnal control. This is the first report of RiceCyc and the analysis is reasonably well described.

We appreciate your encouragement.

Minor Essential Revisions.
It would be better to describe more clearly about the relationships between RiceCyc and other tools such as BiNGO and RiceNet. Apparently it is an advantage that the users can use the RiceCyc knowledge for other tools, but how it is easy or well integrated is not clear.

We appreciate the reviewer's suggestion. We have included additional text in the methods section under 'data analysis' to elaborate on the use of BinGO and RiceNet. All of these tools are designed to work independently and are not integrated. BinGO looks at the enrichment of the Gene Ontology assignments of a given list of genes, *e.g.* in a differentially expressed gene set. RiceNet primarily contains a protein-protein interaction and co-expression network. RiceCyc holds interaction data on protein-metabolite and protein-protein complexes, thus it is tightly associated to the reactions and pathways describing the role of metabolic, and to some extent, transport-related genes. GO annotations can tell, for example, that a gene has an enzyme function-X and plays a role-Y in a biological process, but it does not carry information about 1) the precise step of the pathway/reaction it catalyzes, 2) upstream/downstream reaction components, or 3) interactions with metabolites, ions and other gene products. Also, in the absence of pathways it is difficult to build models for biochemical actions and flux balance analysis. For example, by looking at the gene expression profiles described in the manuscript on the pathways of auxin and serotonin biosynthesis that happens downstream of tryptophan biosynthesis, we built a hypothesis that 1) potentially during the early events of a rice plant challenged by a pathogen, the expression profile of auxin biosynthesis pathway genes are reduced compared to those of the serotonin biosynthesis, thus revealing new approaches to target novel pathways that have not been studied from a disease-resistance perspective and (2) the very same genes in the pathways are also regulated by diurnal photoperiod rhythms. Similar analysis and hypothesis building is less feasible with BinGO and RiceNet datasets/tools. However, we certainly acknowledge that all of these tools complement each other from a systems biology perspective.

The figures are difficult to understand especially about the relationship between gene IDs and expression tables and pathway maps in Figs 2, 4 and 5. It would

be better to use a bigger and clear font for the gene names so that the reader can figure out where the relationship between the gene IDs in the main text and those in the figures. Red outlines are also difficult to figure out.

We appreciate the reviewer's suggestion. The pathway images were enlarged to address the font size concern. The red outlines have been replaced with yellow outlines to improve legibility. The supplementary expression table includes a list of genes mapped to all of the pathways in RiceCyc and is not meant to be compared with individual pathways and figures.

IAA should be spelled out in its first appearance.
There are many "reaction reaction" in the main text and figure legends. Should this be "reaction"?

We have updated the manuscript accordingly.

Level of interest: An article of importance in its field
Quality of written English: Acceptable
